# DRAP: a toolbox for drug response analysis and visualization tailored for preclinical drug testing on patient-derived xenograft models

**DOI:** 10.1186/s12967-019-1785-7

**Published:** 2019-01-29

**Authors:** Quanxue Li, Wentao Dai, Jixiang Liu, Yi-Xue Li, Yuan-Yuan Li

**Affiliations:** 1 School of Biotechnology, East China University of Science and Technology, 130 Meilong Road, Shanhgai, 200237 People’s Republic of China; 2 Shanghai Center for Bioinformation Technology, 1278 Keyuan Road, Shanghai, 201203 People’s Republic of China; 3 Shanghai Industrial Technology Institute, 1278 Keyuan Road, Shanghai, 201203 People’s Republic of China; 4 Shanghai Engineering Research Center of Pharmaceutical Translation & Shanghai Industrial Technology Institute, 1278 Keyuan Road, Shanghai, 201203 People’s Republic of China; 5 Key Lab of Computational Biology, CAS-MPG Partner Institute for Computational Biology, Shanghai Institutes for Biological Sciences, Chinese Academy of Sciences, 320 Yueyang Road, Shanghai, 200031 China

**Keywords:** Patient-derived xenograft, Tumor model, Drug response analysis, Preclinical drug testing, Personalized drug selecting

## Abstract

**Background:**

One of the key reasons for the high failure rate of new agents and low therapeutic benefit of approved treatments is the lack of preclinical models that mirror the biology of human tumors. At present, the optimal cancer model for drug response study to date is patient-derived xenograft (PDX) models. PDX recaptures both inter- and intra-tumor heterogeneity inherent in human cancer, which represent a valuable platform for preclinical drug testing and personalized medicine applications. Building efficient drug response analysis tools is critical but far from adequate for the PDX platform.

**Results:**

In this work, we first classified the emerging PDX preclinical trial designs into four patterns based on the number of tumors, arms, and animal repeats in every arm. Then we developed an R package, DRAP, which implements Drug Response Analyses on PDX platform separately for the four patterns, involving data visualization, data analysis and conclusion presentation. The data analysis module offers statistical analysis methods to assess difference of tumor volume between arms, tumor growth inhibition (TGI) rate calculation to quantify drug response, and drug response level analysis to label the drug response at animal level. In the end, we applied DRAP in two case studies through which the functions and usage of DRAP were illustrated.

**Conclusion:**

DRAP is the first integrated toolbox for drug response analysis and visualization tailored for PDX platform. It would greatly promote the application of PDXs in drug development and personalized cancer treatments.

**Electronic supplementary material:**

The online version of this article (10.1186/s12967-019-1785-7) contains supplementary material, which is available to authorized users.

## Background

It is well known that the major issues in cancer translational medicine include the low success rate of new agents [1, 2] and limited therapeutic benefit of approved drugs in clinical [3, 4]. At present, roughly 90% of preclinical anticancer agents entering clinical trials fail to gain regulatory approval, and the average cost of bringing a new drug to market is over $1 billion [1, 5]. The response rate (RR) is about 10% for cytotoxic agents, and about 30% for targeted agents guided by biomarker test [3, 4]. One of the most frequently cited reasons is the lack of preclinical models that mirror the biology of human tumors, although a diversity of cancer models, such as cell line models and cell line-derived xenograft (CDX) models, have been built and commonly used over late decades [6–8].

In recent years, patient-derived xenograft (PDX) models, which involve directly grafting fresh tumor tissues into immunodeficient mice, have proved to faithfully recapitulate the molecular, genetic, histopathological features of their originating tumors, and particularly represent both inter- and intra-tumor heterogeneity inherent in human cancer [6, 8–12]. It has been widely accepted that PDX models are the most clinically relevant cancer models developed to date [6, 13–16], and the use of PDX platform in drug response study is therefore expanding rapidly.

In spite of the increasing applications of PDX models, PDX drug response data are usually analyzed by tools designed for CDX models [17] or clinical trials [18]. Due to the obvious differences between PDXs and CDXs in both biological properties and experimental techniques, such as genetic heterogeneity and measuring indicators, drug response analysis methods designed for CDXs are essentially not appropriate for PDXs [6]. Similarly, there are noticeable discrepancies between PDX trials and clinical trials, for example, the different criteria for evaluating drug efficacy, the different trial designs regarding sample size, intra-tumor heterogeneity considered or not, which block the application of clinical drug response analysis methods to PDX platform [7, 19]. It is noteworthy that PDX models mimic both inter- and intra-tumor heterogeneity, and thus the PDX drug response experiments could be designed in a more complicated way by which the effects of tumor heterogeneity on drug response can be checked more thoroughly. Hence, it is critical and urgent to select appropriate data analysis methods for preclinical drug testing on PDX platform and develop an integrated toolbox for drug response analysis tailored for PDX platform.

**Fig. 1:**
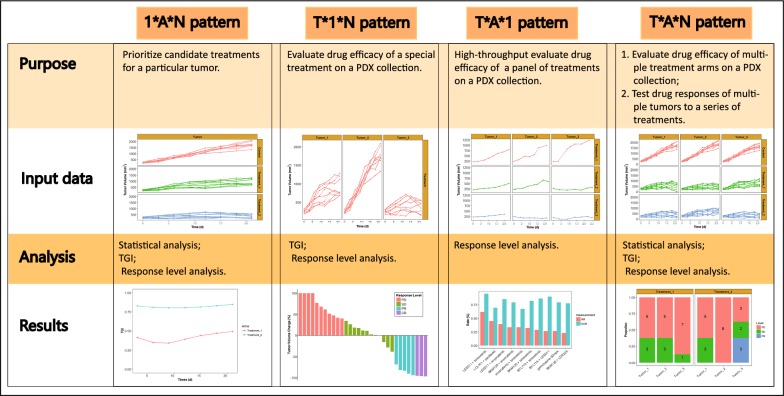
The overview of DRAP. The emerging PDX trial settings could be classified into four patterns: 1*A*N, T*1*N, T*A*1 and T*A*N. Each pattern has its own specific study purpose, and type of input data. DRAP offers three ways to analyze drug response: statistical analysis methods that assess difference of tumor volume between arms are suitable to 1*A*N and T*A*N; tumor growth inhibition (TGI) rate calculation is useful for 1*A*N, T*1*N, and T*A*N; drug response level analysis is suitable to all four patterns. DRAP provides multiple types of tools to present results, including line chart, waterfall plot, and bar diagram

To address this problem, we developed an R package, DRAP, to implement Drug Response Analyses on PDX platform for four typical PDX trial designs. The tools in DRAP involve data visualization, data analysis and conclusion presentation (see Fig. 1 for DRAP overview). Specifically, the data analysis module enables a user to statistically assess the difference of tumor volume between arms, calculate tumor growth inhibition (TGI) rate, and label the drug response at animal level. By applying DRAP to two datasets, an unpublished dataset, and a published one derived from Novartis Institutes for BioMedical Research PDX encyclopedia (NIBR PDXE) [14], the functions of DRAP were demonstrated. We propose that DRAP, the first integrated toolbox for drug response analysis and visualization tailored for preclinical drug testing on PDX platform, would greatly promote the application of PDXs in drug development and personalized cancer treatments.

## Results

### Design of DRAP

After summarizing a series of literatures carrying out drug response studies on PDXs, we classified the emerging PDX preclinical settings into four patterns: 1*A*N, T*1*N, T*A*1 and T*A*N, with the first letter representing the number of *tumors*, the second representing the number of *arms* for each tumor, the third representing the number of *animals* corresponding to one tumor line in each arm. Note that *one* means single and *T/A/N* means multiple. The functions of drug response analysis for each pattern are described as follows (see Fig. 1 for DRAP overview).

1*A*N: This pattern is designed to prioritize candidate treatments for a particular tumor [20]. When PDX avatar models are successfully established and propagated, the animal cohorts are randomized into several arms, with one arm enrolling multiple animals and subjected to a certain treatment or vehicle. Then the volumes of tumor tissues and body weights of animals are measured at a series of time points. DRAP first visualizes the tumor volume data and body weight data for all time points at both the level of individual tumor-bearing mice and the level of single arm. Secondly, DRAP assesses potential differences in tumor volume between arms by using one-way ANOVA, Kruskal–Wallis test, mixed-design ANOVA, linear mixed model (LMM), or permutation strategy, as explained in methods. Then DRAP ranks the arms by calculating tumor growth inhibition (TGI) rate and presents the results of TGI for both end time point and all time points. It is noteworthy that the inter-individual heterogeneity of the animal repeats in a treatment arm at least partly reflects the intra-tumor heterogeneity of the original tumor, which makes it feasible to consider intra-tumor heterogeneity when assessing therapeutic treatments for a tumor. That is, the treatment which leads to significant response in more animals may target more tumor subclones and would show better efficacy when administered to the original tumor. We therefore could prioritize candidate treatments by labeling the drug response level of each animal with complete response (CR), partial response (PR), stable disease (SD) and progressive disease (PD), which are defined based on tumor volume as explained in the section of methods.

T*1*N: This pattern aims to evaluate the anti-tumor efficacy of a particular treatment by using a PDX collection [18, 21, 22]. Since a collection of xenografts are included, the effect of inter-tumor heterogeneity on drug response is sufficiently taken into account. Following the common protocol in preclinical data analysis, the drug response of each tumor line is calculated based on the mean or median of tumor volume values. The setting of multiple animals enrolled in one experimental group helps to increase the accuracy of response level of the tumor, and therefore acquire more precise evaluation of drug efficacy [23]. Still due to the enrollment of multiple animals in each tumor group, intra-tumor heterogeneity could also be considered if needed, similar to 1*A*N pattern.

T*A*1: This pattern is designed for the high-throughput evaluation of a panel of treatment arms [14, 24]. Similar to the above T*1*N pattern, a collection of xenografts are included, therefore inter-tumor heterogeneity is taken into account. As T*A*1 pattern involves a collection of tumors and a panel of treatment arms in one trial, this setting enrolls only one animal in each arm of every tumor line in order to balance costs with outcomes. The performance of this setting has been approved by an independent report [25]. It is noted that since there is only one animal in each arm, intra-tumor heterogeneous response to the same treatment could not be investigated in this setting.

T*A*N: This pattern could be regarded as extended versions of the above three patterns, and could be applied in various situations. While applied for evaluating drug efficacies of multiple treatment arms based on a PDX collection, the analysis is consistent with that of T*1*N pattern [26]. While applied for testing drug responses of multiple tumors to a series of treatments, the analysis is similar to 1*A*N pattern [17]. Of note, since this pattern includes multiple tumors in one trial and multiple animals in each arm, it allows for the investigation of both inter-tumor heterogeneity and intra-tumor heterogeneity.

For the experimental patterns mentioned above, 1*A*N, T*1*N, T*A*1 and T*A*N, DRAP offers functions to assess difference of tumor volume across arms, calculate TGI for each arm, label drug response level of animals, calculate response evaluation index of treatment arms, and visualize the analysis results.

### Case study 1: 1*A*N pattern

**Fig. 2:**
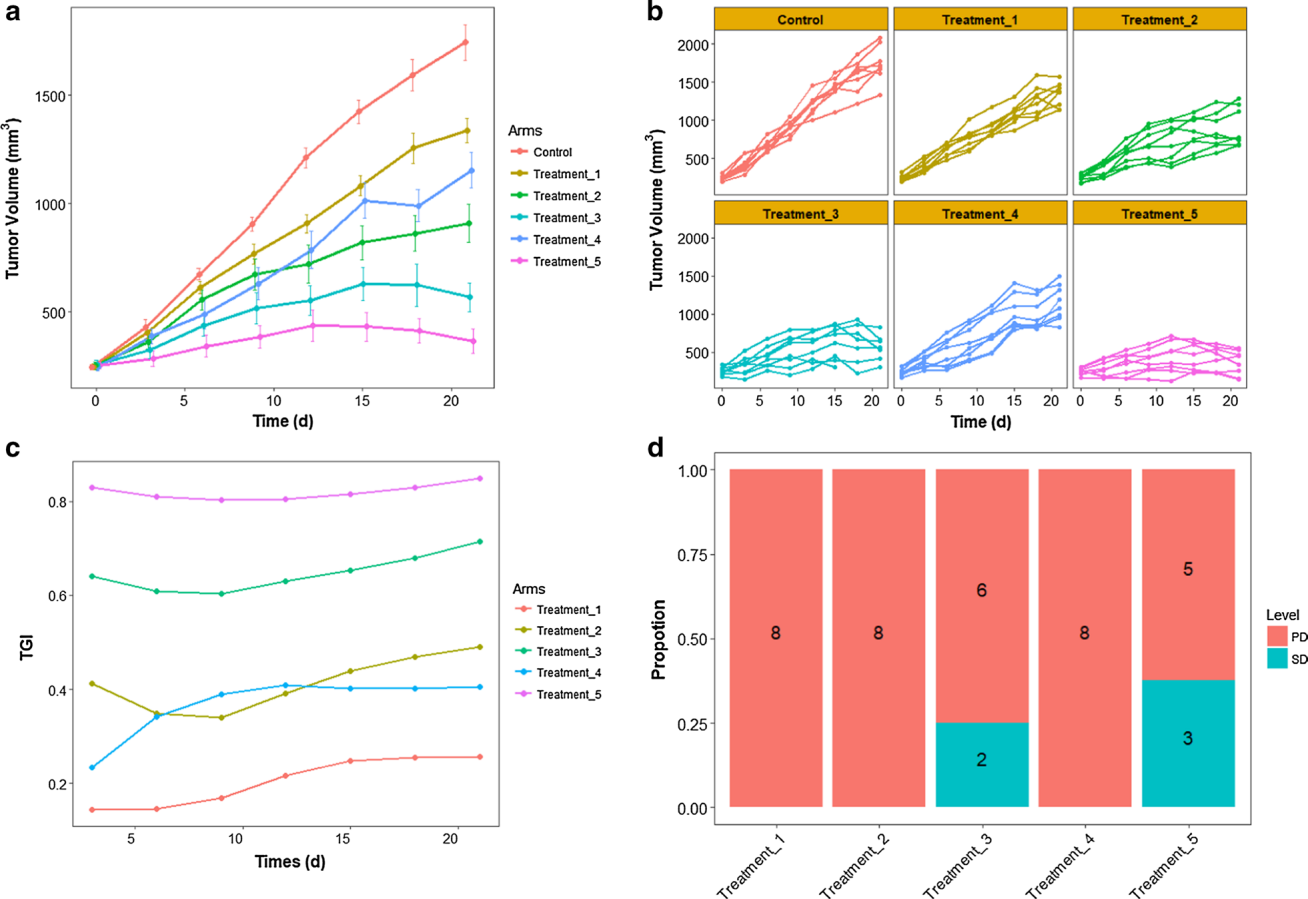
The analysis and visualization of 1*A*N pattern. **a** Tumor volume data at the level of arm (mean ± SEM). **b** Tumor volume data at the level of animal. **c** TGI value at all timepoints. **d** Drug response level of each arm

One of our unpublished datasets, generated from preclinical drug response study on PDX platform, were adopted to demonstrate the functions of DRAP for 1*A*N pattern. The dataset involves five treatment arms and one vehicle, with each arm enrolling eight animals. Tumor volumes and body weights of every animal were measured every 3 days. The drug administration lasted for 3 weeks. By using DRAP, the tumor volume data and animal body weight data were visualized at both the level of individual tumor-bearing mice and the level of single arm for all time points, which eases the interpretation of the data and allows judgement of kinetics [27] (Fig. 2a, b). Body weight data was also presented in the same way (Additional file 1: Figs S4 and S5). Besides, DRAP also offers functions to calculate and present the relative change of tumor volume and animal body weight based on the initial baseline for each animal (Additional file 1: Figs S6 and S7).

To assess potential differences in tumor volume between arms, one-way ANOVA, Kruskal–Wallis test, mixed-design ANOVA, linear mixed model (LMM) and permutation test were used independently. The results shown that there was significant difference between arms given all methods (see results in supplement). Furthermore, LMM and permutation test indicated that all treatment arms were significantly different from vehicle.

TGI of every arm was calculated for each time point and presented in Fig. 2c, which showed the extent of tumor inhibition during treatment. It was indicated that *treatment_5* has the best efficacy among the five candidates.

The response levels for each animal were defined by the method *NPDXE.Response* [14], as illustrated in supplement. There are three animals with level stable disease (SD) in *treatment_5* arm and two animals with level SD in *treatment_3* arm (Fig. 2d). The response evaluation index of every arm were calculated based on the response level of each animal. In summary of these analysis results, *treatment_5* has the best efficacy among the five candidates (see details in Additional file 1). Through the PDX trial study, the optimal treatment for the patient is *treatment_5*.

### Case study 2: T*A*1 pattern

We use the dataset derived from Novartis Institutes for BioMedical Research PDX encyclopedia (NIBR PDXE) as example to introduce the function of DRAP for T*A*1 pattern [14]. The dataset includes both tumor volume and body weight data for 6 tumor types, 277 tumors, and total 4771 animals responded to 61 treatments. The dataset used here includes information “Model”, “Tumor Type”, “Treatment”, “Days Post T0”, and “Volume”.

The response level of every animal is labeled by using the method *NPDXE.Response* [14]. Based on the response level of each animal, multiple purposes can be realized, including ranking drug efficacy of all arms in a special type of tumor, evaluating drug efficacy of a special treatment in different types of tumors or in a special type of tumor. DRAP offers analysis and visualization tools for these purposes.

**Fig. 3:**
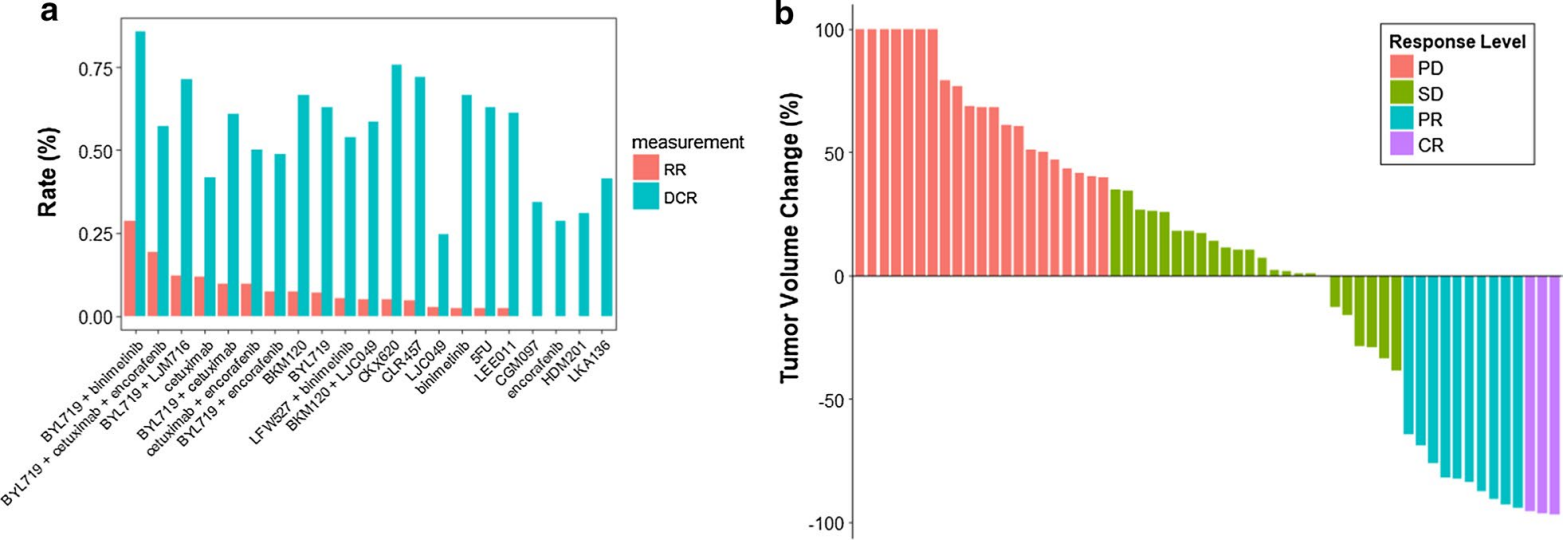
The analysis and visualization of T*A*1 pattern. **a** The drug efficacy of all arms in CRC. **b** The waterfall plot of each animal response to the combination of BYL719 and LJM716 in GC

The data of colorectal cancer (CRC) in NIBR PDXE was used to illustrates the functions of DRAP of ranking drug efficacy of all arms in a special type of tumor. DRAP ranks and presents the drug efficacy of all arms for CRC as in Fig. 3a. The results showed that the combination of BYL719 and Binimetinib has the best efficacy, with RR being 28.57% and DCR being 85.71%. BYL719 is a selective inhibitor of PI3Kalpha [28]. LJM716 is an antibody drug targeted HER3 [29]. The information could help to determine the best candidate drugs or drug combinations for CRC in clinical trial.

For a new treatment, the question most frequently encountered is how to choose the preferred indication for clinical trial. To solve this question with PDXs, drug efficacy of the new treatment in different types of tumors should be evaluated and ranked. This part illustrates this function of DRAP with the data of different types of tumors respond to the combination of BYL719 and LJM716 in NIBR PDXE. Among the six tested tumor types, this treatment shown highest efficacy in gastric cancer (GC) (Additional file 1: Fig S12). Based on this information, the developers could choose GC as the preferred indication for this new treatment in clinical trial.

While evaluating drug efficacy of a special treatment in a particular type of tumor, waterfall plots is efficient way to represent the response level of individual animal, which provide an ease of visualization and interpretation [27]. This part illustrates this function of DRAP with the data of GC respond to the combination of BYL719 and LJM716 in NIBR PDXE. As in Fig. 3b, we could easily get the information of drug efficacy for each animal, how many animals in each response level, and the total drug efficacy in all samples.

## Discussion

Along the development of preclinical models in the last four decades, the use of preclinical models has become more and more routine in almost every aspect of cancer research [6], among which PDX models so far are the most clinically relevant cancer models [13–16]. Several big PDX repositories have been built both in academic and industrial communities, such as EuroPDX (https://www.europdx.eu/), CrowBio (https://www.crownbio.com/), Patient-Derived Models Repository (PDMR) (https://pdmr.cancer.gov/) and NIBR PDXE [14]. Simultaneously, the number of articles related to “patient-derived xenograft” has greatly exploded in PubMed in recent years. That is, the use of PDX platform in drug response study is increasing rapidly.

In the area of drug response study, there have been quite a few data analysis tools developed for specific platforms. For cancer cell line models, drc [30], GRcalculator [31, 32], GraphPad (http://www.graphpad.com), and so on, calculate effective dose of drug based on cell growth inhibition. For clinical trials, Response Evaluation Criteria in Solid Tumors (RECIST) [33, 34] labels response level for each patient based on tumor volume change during certain time period. It is noted that response surface models could be applied to seek the best dose for drug combination in both cancer cell models and clinical trials [35]. Besides, methods for predicting drug response based on omics data have rose in recent years [36–38]. In this sense, it is critical and urgent to build efficient data analysis toolbox specific for preclinical drug testing on PDX platform.

PDX models have been applied in multiple trial settings, including drug selecting for a particular patient, preclinical drug efficacy evaluation for a new treatment or a panel of treatments. Each setting need specific data analysis strategy and result presentation. It is noticeable that PDX models mimic both inter- and intra-tumor heterogeneity, and thus the effects of tumor heterogeneity on drug response can be checked in greater detail on PDX platform. Taken together, these make drug response analysis of PDX experiments much more complex than that of cell line based experiments.

In the current work, we developed an integrated toolbox, DRAP, to carry out drug response analysis and visualization tailored for preclinical drug testing on PDX models. DRAP accommodates four typical PDX preclinical trial settings, with each setting corresponding to a specific context of application. Compared with cancer cell lines, PDX models recapitulate tumor heterogeneity of both inter- and intra-tumor. Compared with clinical trials, PDX models could be used to test multiple drugs for one tumor simultaneously; moreover, the diverse responses in one arm represent intra-tumor heterogeneity to a certain extent. In order to serve various study purposes, different analysis strategies and methods are required. Our R package enables users to implement data visualization, drug response analysis and conclusion presentation smoothly for four commonly used PDX trial settings.

Compared with the existing drug response analysis methods for PDX models, DRAP integrated three ways to study drug response: statistical analysis methods to assess difference of tumor volume between arms, tumor growth inhibition (TGI) rate calculation to quantify drug response, and drug response level analysis to label drug response at animal level. These methods could be used separately or jointly. Besides, DRAP provides tools to visualize data and present conclusion in flexible ways, such as line chart for presenting TGI of different arms, bar diagram for presenting drug response level among different arms, and waterfall plot for presenting the response level of every tumor.

DRAP has the space to be improved. For example, drug response analysis of the current version is based on tumor volume data, so drug response data for hematologic tumors could not be adopted by DRAP although PDX models for hematologic tumors have been successfully established and been used for preclinical study [39, 40]; similarly, drug response data generated by bioluminescence could not be handled by DRAP [17]. Additionally, DRAP adopted three existing methods to label drug response level [14, 18, 25], all of which differ in the criteria for initial tumor volume, tumor volume changes during drug administration, and the time of duration. When the standard for labeling response level on PDX platform is set up, it should be integrated into our toolbox.

## Conclusion

DRAP is the first integrated toolbox for drug response analysis and visualization tailored for preclinical drug testing on PDX models. It offers practical tools to visualize data, analyze data, and present conclusion. Particularly, the effects of inter- and intra-tumor heterogeneity on drug response can be estimated via DRAP. It would greatly promote the application of PDXs in drug development and personalized cancer treatments. It is flexible and extendable to perform advanced data analysis in the field of precision medicine.

## Methods

### Statistical analysis of tumor volume

Several statistical methods were adopted to assess potential differences of tumor volumes across arms, including conventional ANOVA [41, 42], Kruskal–Wallis test [43], Scheirer–Ray–Hare test [44], mixed-design ANOVA [18], linear mixed model (LMM) [45], and permutation test [46, 47].

In general, conventional ANOVA is used to analyze tumor volume data measured at the end of experiment, which is borrowed from drug response data analysis for CDX models where the tumor volumes at the starting time point tend to be consistent across animals due to the homogeneity of cell lines. Therefore, this method by analogy applies to PDX based experiments only when the tumor volumes at the starting point do not significantly differ among animals. However, for the sake of the dramatic heterogeneity of tumor tissue, the growth rate of tumor in PDX model could be greatly different among the animal cohort after tumor tissue implanting [48]. This would lead to significant difference in tumor volumes of different animals at starting point of drug treatment. To address this problem, we integrated the data of tumor growth rate during treatment into ANOVA method, and in this way the tumor volumes could be rectified.

Because the tumor volume of each animal is repeatedly measured at several timepoints, repeated analysis methods including mixed-design ANOVA and linear mixed model (LMM) are also offered, both of which have been applied to analyze drug response data generated from PDX experiments [18, 45].

Besides the above parametric statistical analysis, the corresponding nonparametric statistical analysis methods of one-way ANOVA and two-way ANOVA, Kruskal–Wallis test [43] and Scheirer–Ray–Hare test [44], are provided to analyze the tumor volume of end point and the tumor growth rate.

The permutation strategy is adopted to test whether significant difference in the tumor volume growth curves exists between different arms. This method is similar to the function of *compareGrowthCurves* in statmod package [46, 47]. For each pair of arms, DRAP first calculates t-statistics or Wilcox-statistics for each time point, and then calculates the mean of statistics among all time points. Subsequently, the animals in the arm pair are randomly allocated to two arms and the mean statistics was recalculated for 1000 times. The P value is the proportion of permutations where the mean statistics is greater in absolute value than the mean statistics for the original data set. Each pair of arms generates a P value. At last, the P-values are adjusted based on multiple testing among all possible arm pairs.

### Calculation of tumor growth inhibition rate

Tumor growth inhibition (TGI) rate is one of the most commonly used metrics to quantify the drug response of treatment arms compared to the control arm. The basic way to calculate TGI is following:

1 $${\text{TGI}} = 1 - \frac{{F\left( {{\text{V}}_{\text{T}} } \right)}}{{F\left( {{\text{V}}_{\text{C}} } \right)}}*100\%$$

*F*(V_T_) and *F*(V_C_) denote the calculating ways for the treatment arm and control arm respectively. We provides three types of *F* function to calculate TGI: (1) *F* = V_t_ − V_0_ [49]; (2) *F* = V_t_/V_0_ [50]; (3) *F* = area under the curve of tumor volume (AUC) [51]. V_t_ and V_0_ represent the mean tumor volume at the time t and time 0 respectively. For example, if *F* = V_t_ − V_0_, the TGI is expression as:

2 $${\text{TGI}} = 1 - \frac{{{\text{V}}_{{{\text{T}},{\text{t}}}} - {\text{V}}_{{{\text{T}},0}} }}{{{\text{V}}_{{{\text{C}},{\text{t}}}} - {\text{V}}_{{{\text{C}},0}} }}*100\%$$

where V_T,t_ and V_T,0_ represent the mean tumor volume of treatment arm at the time t and time 0 respectively, V_C,t_ and V_C,0_ represent the mean tumor volume of control arm at the time t and time 0 respectively.

### Labeling of drug response level

Drug response level is calculated to label the drug response of each animal, such as complete response (CR), partial response (PR), stable disease (SD) and progressive disease (PD) [14, 17, 18, 52]. The level is defined according to the tumor volume change after treatment. We implemented three standards to label drug response level: the one built in Novartis Institutes for BioMedical Research PDX encyclopedia (*NPDXE.Response*) [14], the one in Pediatric Preclinical Testing Program (*PPTP.Response*) [25, 50], and the one based on the relative change of tumor volumes (*RC.Response*) [18]. The details of the three methods could be found in supplement. Considering that tumor growth is influenced by the strains of mice, DRAP enables users to adjust the standards for defining response levels according to the experimental data and practical needs.

After labeling response level for each animal, the response evaluation indexes for each arm is calculated, including response rate (RR) and disease control rate (DCR). RR is the proportion of CR and PR among all testing objects in one arm, and DCR is the proportion of CR, PR and SD.

## Additional file

**Additional file 1.** User’s guide for DRAP.

## Abbreviations

PDX: patient-derived xenograft
DRAP: drug response analysis on PDX models
TGI: tumor growth inhibition
RR: response rate
DCR: disease control rate
CR: complete response
PR: partial response
SD: stable disease
PD: progressive disease
CRC: colorectal cancer
GC: gastric cancer
NIBR PDXE: Novartis Institutes for BioMedical Research PDX encyclopedia
