# Supplementary material for: DRAP: a toolbox for drug response analysis and visualization tailored for preclinical drug testing on patient-derived xenograft models
Source: J Transl Med. 2019 Jan 29;17:39. doi: 10.1186/s12967-019-1785-7 (PMC6350365; doi:10.1186/s12967-019-1785-7)
Supplement: Supplementary file 1 — Additional file 1. User’s guide for DRAP. [file 12967_2019_1785_MOESM1_ESM.docx]

DRAP: a toolbox for drug response analysis and visualization tailored for preclinical drug testing on patient-derived xenograft models

User’s Guide

First edition: 15 January 2018

Last revised: 12 September 2018

Contents

[1. Introduction 2](#_Toc518309190)

[2. Design 2](#_Toc518309191)

[3. 1*A*N pattern 4](#_Toc518309192)

[3.1 Input data 4](#_Toc518309193)

[3.2 Data presentation 6](#_Toc518309194)

[3.2.1 Presenting the volume data 7](#_Toc518309195)

[3.2.2 Presenting the body weight data 8](#_Toc518309196)

[3.2.3 Presenting the relative change of data 9](#_Toc518309197)

[3.3 Statistical analysis 11](#_Toc518309198)

[3.3.1 Statistical test 11](#_Toc518309199)

[3.3.2 Permutation test 13](#_Toc518309200)

[3.4 TGI 13](#_Toc518309201)

[3.5 Response level analysis 16](#_Toc518309202)

[4. T*1*N pattern 19](#_Toc518309203)

[5. T*A*1 pattern 19](#_Toc518309204)

[5.1 Input data 20](#_Toc518309205)

[5.2 Response level analysis 20](#_Toc518309206)

[5.2.1 Ranking drug efficacy of all arms in one type of tumor 21](#_Toc518309207)

[5.2.2 Evaluating drug efficacy of one arm in all types of tumors 22](#_Toc518309208)

[5.2.3 Evaluating drug efficacy of one arm in one type of tumor 23](#_Toc518309209)

[6. T*A*N pattern 24](#_Toc518309210)

[6.1 Input data 25](#_Toc518309211)

[4.2 Data presentation and analysis 25](#_Toc518309212)

[4.3 Response level analysis 28](#_Toc518309213)

[Reference 31](#_Toc518309214)

## 1. Introduction

Patient-derived xenograft (PDX) models recapitulate the molecular, genetic, histopathological features of their originating tumors, and particularly represent both inter- and intra-tumor heterogeneity inherent in human cancer [1-6]. it has been widely accepted that PDX models are the most clinically relevant cancer models developed to date [2, 7-10], and the use of PDX platform in drug response study is therefore expanding rapidly. In spite of this, PDX drug response data are usually analyzed by the tools designed for CDX models [11] or clinical trials [12]. Due to the obvious differences between PDXs and CDXs in both biological properties and experimental techniques, such as genetic heterogeneity and measuring indicators, drug response analysis methods designed for CDXs are actually not appropriate for PDXs [2]. Similarly, there are noticeable discrepancies between PDX trials and clinical trials, for example, the different criteria for evaluating drug efficacy, the different trial designs regarding sample size, intra-tumor heterogeneity considered or not, which block the application of clinical drug response analysis methods to PDX platform [13, 14]. It is noteworthy that PDX models mimic both inter- and intra-tumor heterogeneity, and thus the PDX drug response experiments could be designed in a more complicated way by which the effects of tumor heterogeneity on drug response can be checked more thoroughly. Hence, it is critical and urgent to develop integrated analysis tools tailored for preclinical drug testing on PDX platform [15]. In the current work, we developed an R package DRAP, which implements Drug Response Analyses on PDX platform separately for the four patterns, involving data visualization, data analysis and conclusion presentation for four types of PDX trial settings. The data analysis module offers statistical analysis methods to assess difference of tumor volume between arms, tumor growth inhibition (TGI) rate calculation to quantify drug response, and drug response level analysis to label the drug response for each animal. We propose that DRAP would greatly promote the application of PDXs in drug development and personalized cancer treatments.

## 2. Design

After summarizing a series of literatures carrying out drug response studies on PDXs, we classified the emerging PDX preclinical settings into four patterns: *1*A*N, T*1*N*, *T*A*1* and *T*A*N*, with the first letter representing the number of *tumors*, the second representing the number of *arms* for each tumor, the third representing the number of *animals* corresponding to one tumor line in each arm. Note that *one* means single and *T/A/N* means multiple. The functions of drug response analysis for each pattern are described as follows (see figure 1 for DRAP overview).


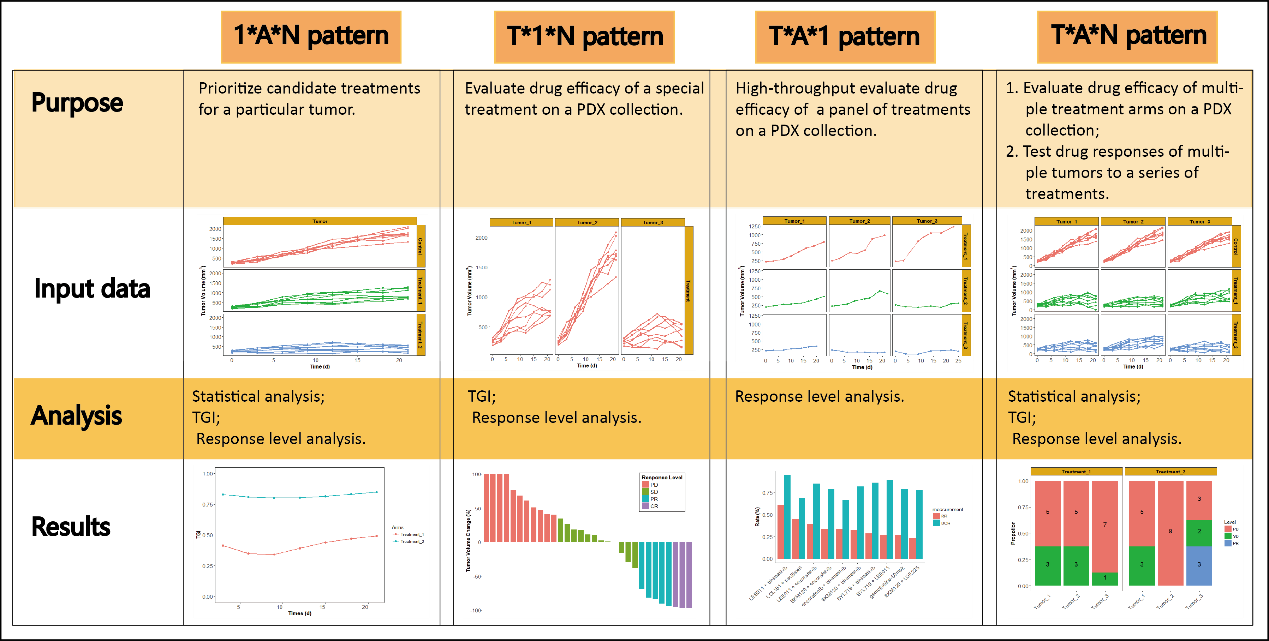


Figure S1. The overview of DRAP. The emerging PDX trial settings could be classified into four patterns: *1*A*N, T*1*N*, *T*A*1* and *T*A*N*. Each pattern has its own specific study purpose, and type of input data. DRAP offers three ways to analyze drug response: statistical analysis methods that assess difference of tumor volume between arms are suitable to *1*A*N* and *T*A*N*; tumor growth inhibition (TGI) rate calculation is useful for *1*A*N, T*1*N*, and *T*A*N*; drug response level analysis is suitable to all four patterns. DRAP provides multiple types of tools to present results, including line chart, waterfall plot, and bar diagram.

*1*A*N*: This pattern is designed to prioritize candidate treatments for a particular tumor [16]. When PDX avatar models are successfully established and propagated, the animal cohorts are randomized into several arms, with one arm enrolling multiple animals and subjected to a certain treatment or vehicle. Then the volumes of tumor tissues and body weights of animals are measured at a series of time points. DRAP first visualizes the tumor volume data and body weight data for all time points at both the level of individual tumor-bearing mice and the level of single arm. Secondly, DRAP assesses potential differences in tumor volume between arms by using one-way ANOVA, Kruskal-Wallis test, mixed-design ANOVA, linear mixed model (LMM), or permutation strategy, as explained in methods. Then DRAP ranks the arms by calculating tumor growth inhibition (TGI) rate and presents the results of TGI for both end time point and all time points. It is noteworthy that the inter-individual heterogeneity of the animal repeats in a treatment arm at least partly reflects the intra-tumor heterogeneity of the original tumor, which makes it feasible to consider intra-tumor heterogeneity when assessing therapeutic treatments for a tumor. That is, the treatment which leads to significant response in more animals may target more tumor subclones and would show better efficacy when administered to the original tumor. We therefore could prioritize candidate treatments by labeling the drug response level of each animal with complete response (CR), partial response (PR), stable disease (SD) and progressive disease (PD), which are defined based on tumor volume as explained in the section of methods.

*T*1*N*: This pattern aims to evaluate the anti-tumor efficacy of a particular treatment by using a PDX collection [12, 17, 18]. Since a collection of xenografts are included, the effect of inter-tumor heterogeneity on drug response is sufficiently taken into account. Following the common protocol in preclinical data analysis, the drug response of each tumor line is calculated based on the mean or median of tumor volume values. The setting of multiple animals enrolled in one experimental group helps to increase the accuracy of response level of the tumor, and therefore acquire more precise evaluation of drug efficacy [15]. Still due to the enrollment of multiple animals in each tumor group, intra-tumor heterogeneity could also be considered if needed, similar to *1*A*N* pattern.

*T*A*1*: This pattern is designed for the high-throughput evaluation of a panel of treatment arms [8, 19]. Similar to the above *T*1*N* pattern, a collection of xenografts are included, therefore inter-tumor heterogeneity is taken into account. As *T*A*1* pattern involves a collection of tumors and a panel of treatment arms in one trial, this setting enrolls only one animal in each arm of every tumor line in order to balance costs with outcomes. The performance of this setting has been approved by an independent report [20]. It is noted that since there is only one animal in each arm, intra-tumor heterogeneous response to the same treatment could not be investigated in this setting.

*T*A*N*: This pattern could be regarded as extended versions of the above three patterns, and could be applied in various situations. While applied for evaluating drug efficacies of multiple treatment arms based on a PDX collection, the analysis is consistent with that of *T*1*N* pattern [21]. While applied for testing drug responses of multiple tumors to a series of treatments, the analysis is similar to *1*A*N* pattern [11]. Of note, since this pattern includes multiple tumors in one trial and multiple animals in each arm, it allows for the investigation of both inter-tumor heterogeneity and intra-tumor heterogeneity.

For the experimental patterns mentioned above, *1*A*N, T*1*N*, *T*A*1* and *T*A*N*, DRAP offers functions to assess difference of tumor volume across arms, calculate TGI for each arm, label drug response level of animals, calculate response evaluation index of treatment arms, and visualize the analysis results.

## 3. 1*A*N pattern

This pattern is designed to prioritize candidate treatments for a particular tumor [16]. When PDX avatar models are successfully established and propagated, the animal cohorts are randomized into several arms, with one arm enrolling multiple animals and subjected to a certain treatment or vehicle.

### 3.1 Input data

One of our unpublished datasets were adopted to demonstrate the function of DRAP for *1*A*N pattern*. The dataset involves five treatments and one vehicle, with each arm enrolling eight animals. Tumor volume and body weight of each animal were measured every three days. The drug administration lasted for three weeks.

The input data of DRAP for this pattern includes tumor volume data or animal body weight data. Tumor volume data must at least include the columns “*Arms*” “*ID*” “*Times*” and “*Volume*”; animal body weight data must at least include the columns “*Arms*” “*ID*” “*Times*” and “*BodyWeight*”.

data(oneAN.volume.data)

oneAN.volume.data[1:10,]

Arms ID Times Volume

1 Control mouse_02 0 238.0

2 Control mouse_02 3 576.2

3 Control mouse_02 6 675.3

4 Control mouse_02 9 1051.0

5 Control mouse_02 12 1458.4

6 Control mouse_02 15 1552.2

7 Control mouse_02 18 1870.2

8 Control mouse_02 21 2084.4

9 Control mouse_03 0 223.3

10 Control mouse_03 3 427.8

data(oneAN.bw.data)

oneAN.bw.data[1:10,]

Arms ID Times BodyWeight

1 Control mouse_02 0 14.96

2 Control mouse_02 3 14.29

3 Control mouse_02 6 13.98

4 Control mouse_02 9 14.20

5 Control mouse_02 12 14.45

6 Control mouse_02 15 14.39

7 Control mouse_02 18 14.02

8 Control mouse_02 21 14.24

9 Control mouse_03 0 16.87

10 Control mouse_03 3 16.86

The input data is summarized by *DataSummary*, including animal numbers, mean, standard derivation, and standard error of tumor volumes or body weight in every arm at each time. Besides, this function also checks whether the input data involves the required information.

oneAN.v.s <- DataSummary(data = oneAN.volume.data,

type = 'Volume',

pattern = 'oneAN',

measure.var = 'Volume',

group.vars = c('Arms','Times'))

oneAN.v.s [1:10,]

Arms Times N Volume SD SE

1 Control 0 8 244.2500 35.72382 12.63028

2 Control 3 8 430.3875 102.04799 36.07941

3 Control 6 8 675.0375 77.67710 27.46300

4 Control 9 8 907.1875 90.63286 32.04356

5 Control 12 8 1211.7750 134.44495 47.53347

6 Control 15 8 1424.8250 153.15051 54.14688

7 Control 18 8 1593.4875 208.38244 73.67432

8 Control 21 8 1743.9625 233.85203 82.67918

9 Treatment_1 0 8 245.3625 39.34586 13.91086

10 Treatment_1 3 8 404.8000 72.83029 25.74940

oneAN.bw.s <- DataSummary(data = oneAN.bw.data,

type = 'BodyWeight',

pattern = 'oneAN',

measure.var = 'BodyWeight',

group.vars = c('Arms','Times'))

oneAN.bw.s [1:10,]

Arms Times N BodyWeight SD SE

1 Control 0 8 16.69625 1.1072609 0.3914759

2 Control 3 8 16.92625 1.4752427 0.5215771

3 Control 6 8 16.73000 1.3606721 0.4810702

4 Control 9 8 16.53250 1.2702615 0.4491053

5 Control 12 8 16.46625 1.5606586 0.5517761

6 Control 15 8 16.27000 1.5011424 0.5307340

7 Control 18 8 16.10875 1.4986035 0.5298364

8 Control 21 8 16.17125 1.4315470 0.5061283

9 Treatment_1 0 8 16.23500 0.8150197 0.2881530

10 Treatment_1 3 8 16.25625 1.0996615 0.3887890

### 3.2 Data presentation

For this pattern, two ways are offered to present the tumor volume data or animal body weight data: for every arm or for every animal. While presenting data for every arm, DRAP shows mean ± SEM at every time point. Tumor volume data is presented by *plotVolumeGC*. Body weight data is presented by *plotBWeigthGC*. The presentation of relative change of tumor volume and body weight is implemented by *plotRC*.

#### 3.2.1 Presenting the volume data

While presenting tumor volume data at the level of animal, each curve represents the tumor volume change of one animal, and the curves are grouped by arms (Figure S2).

plotVolumeGC(oneAN.volume.data, level = 'Animal', pattern = 'oneAN')


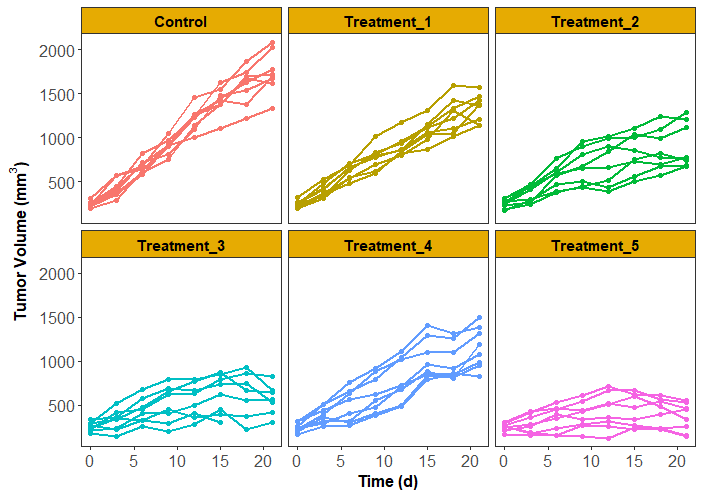


Figure S2. The presentation of tumor volume data at the level of animal.

While presenting tumor volume data at the level of arm, each curve represents the change of the mean tumor volume of each arm (Figure S3).

plotVolumeGC(oneAN.volume.data, level = 'Arm', pattern = 'oneAN', position.dodge = 0.5)


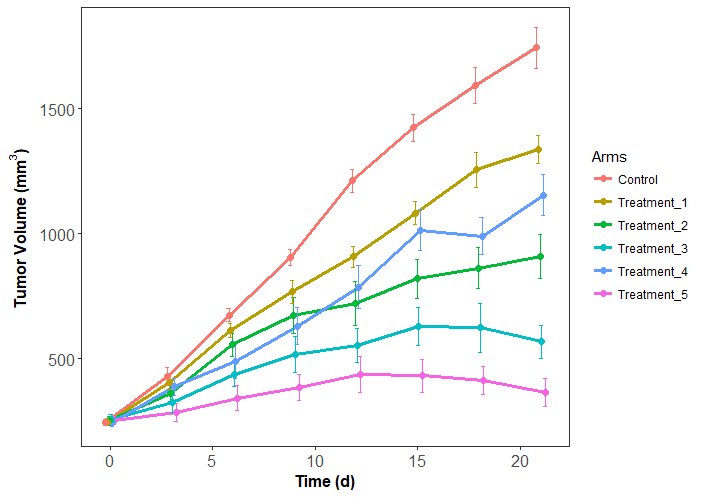


Figure S3. The presentation of tumor volume data at the level of arm.

#### 3.2.2 Presenting the body weight data

While presenting body weight data at the level of animal, the form is like the presentation of tumor volume data at the level of animal (Figure S4).

plotBWeightGC(oneAN.bw.data, level = 'Animal', pattern = 'oneAN')


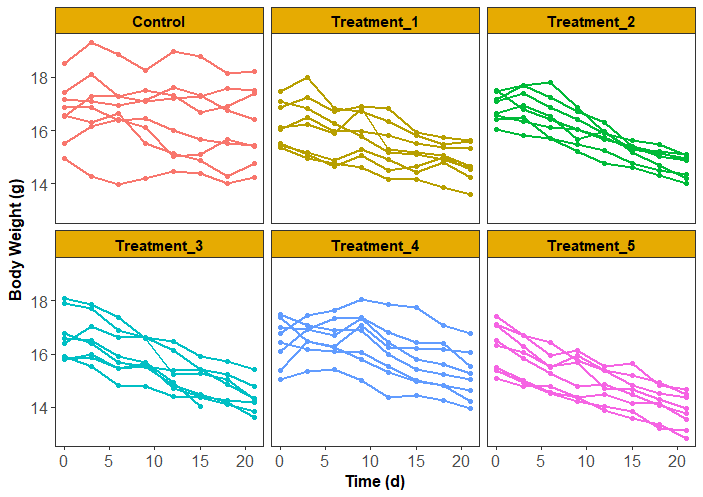


Figure S4. The presentation of body weight data at the level of animal.

While presenting body weight data at the level of arm, the form is like the presentation of tumor volume data at the level of arm (Figure S5).

plotBWeightGC(oneAN.bw.data, level = 'Arm', pattern = 'oneAN', position.dodge = 0.5)


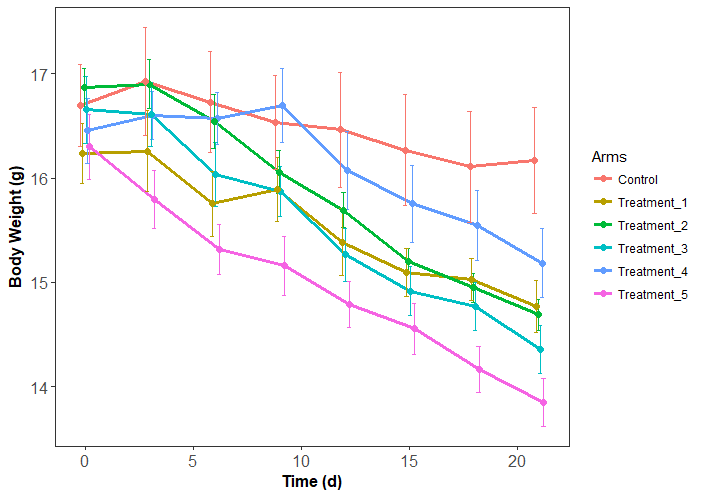


Figure S5. The presentation of body weight data at the level of arm.

#### 3.2.3 Presenting the relative change of data

Besides the presentation of primary tumor volume data and animal body weight data, DRAP also offers functions to calculate and present the relative change of tumor volume and animal body weight based on the initial baseline for each animal. Relative change is calculated by *RelativeChange*, and presented by *plotRC*. Figure S6 and Figure S7 show the relative change of tumor volume data and body weight data respectively.

oneAN.v.rc <- RelativeChange(oneAN.volume.data, type = 'Volume',rm.baseline = T)

head(oneAN.v.rc)

Arms ID Times RelativeChange

1 Treatment_3 mouse_01 0 0.000000

2 Treatment_3 mouse_01 12 94.676806

3 Treatment_3 mouse_01 15 115.852588

4 Treatment_3 mouse_01 18 118.338696

5 Treatment_3 mouse_01 21 56.361509

6 Treatment_3 mouse_01 3 7.429073

plotRC(data = oneAN.volume.data, type = 'Volume', pattern = 'oneAN')


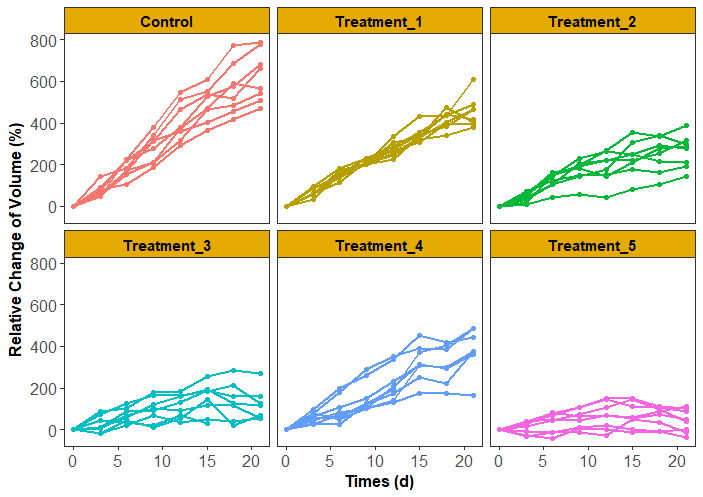


Figure S6. The presentation for the relative change of tumor volume data.

plotRC(data = oneAN.bw.data, type = 'BodyWeight', pattern = 'oneAN')


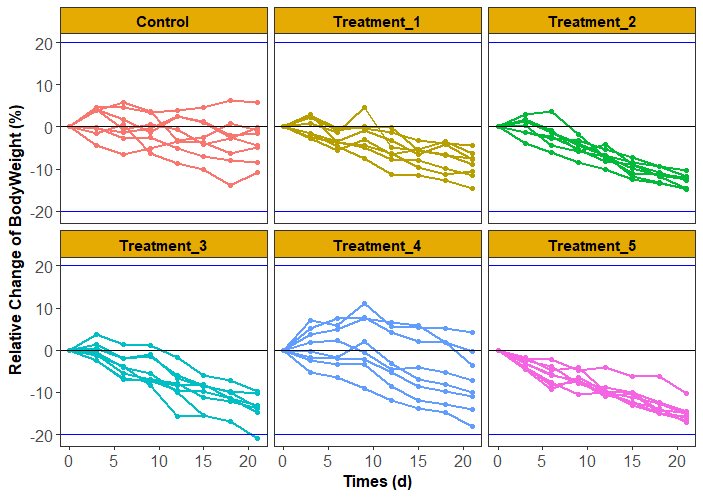


Figure S7. The presentation for the relative change of body weight data.

Since the animal ethics requires that the relative change of body weight of animal cannot exceed more than 20% in experiment, we add the reference lines at the relative change of 20% in Figure S7.

### 3.3 Statistical analysis

To assess potential differences in tumor volume between arms, DRAP offers several statistical methods, including conventional ANOVA, Kruskal-Wallis test, mixed-design ANOVA [12], linear mixed model (LMM) [22], and permutation test [23, 24]. Except permutation test, all other methods are included in function *DRAnalysis*. Permutation test is implemented by *CompareGC*. Users need to choose the appropriate method based the on the characteristics of the data and the applicable conditions of these methods in statistics.

#### 3.3.1 Statistical test

In general, conventional ANOVA is used to analyze tumor volume data measured at the end of experiment, which is borrowed from drug response data analysis for CDX models where the tumor volumes at the starting time point tend to be consistent across animals due to the homogeneity of cell lines. Therefore, this method by analogy applies to PDX based experiments only when the tumor volumes at the starting point do not significantly differ among animals. The output of this method is as following.

DRAnalysis(oneAN.volume.data, pattern = 'oneAN', method = 'endpoint.ANOVA')

Df Sum Sq Mean Sq F value Pr(>F)

Arms 5 10116245 2023249 47.77 4.84e-16 ***

Residuals 41 1736514 42354

---

Signif. codes: 0 ‘***’ 0.001 ‘**’ 0.01 ‘*’ 0.05 ‘.’ 0.1 ‘ ’ 1

However, for the sake of the dramatic heterogeneity of tumor tissue, the growth rate of tumor in PDX model could be greatly different among the animal cohort after tumor tissue implanting [25]. This would lead to significant difference in tumor volumes of different animals at starting point of drug treatment. To address this problem, we integrated the data of tumor growth rate during treatment into ANOVA method, and in this way the tumor volumns could be rectified. The output of this method is as following.

DRAnalysis(oneAN.volume.data, pattern = 'oneAN', method = 'GR.ANOVA')

Df Sum Sq Mean Sq F value Pr(>F)

Arms 5 24336 4867 58.72 <2e-16 ***

Residuals 42 3482 83

---

Signif. codes: 0 ‘***’ 0.001 ‘**’ 0.01 ‘*’ 0.05 ‘.’ 0.1 ‘ ’ 1

Because the tumor volume of each animal is repeatedly measured at several timepoints, repeated analysis methods including mixed-design ANOVA and linear mixed model (LMM) are also offered, both of which have been applied to analyze drug response data generated from PDX experiments [12, 22]. The output of method ‘mixed-design ANOVA’ is as following.

DRAnalysis(oneAN.volume.data, pattern = 'oneAN', method = 'mixed.ANOVA')

Error: ID

Df Sum Sq Mean Sq F value Pr(>F)

Arms 5 18058977 3611795 22.54 6.48e-11 ***

Residuals 42 6731052 160263

---

Signif. codes: 0 ‘***’ 0.001 ‘**’ 0.01 ‘*’ 0.05 ‘.’ 0.1 ‘ ’ 1

Error: ID:Times

Df Sum Sq Mean Sq F value Pr(>F)

Residuals 48 36685441 764280

Error: Within

Df Sum Sq Mean Sq F value Pr(>F)

Residuals 286 2047341 7159

The output of method ‘LMM’ is as following.

DRAnalysis(oneAN.volume.data, pattern = 'oneAN', method = 'LMM')

Value Std.Error DF t-value p-value

(Intercept) 1028.8641 50.06704 334 20.549729 3.089150e-61

ArmsTreatment_1 -201.4625 70.80549 42 -2.845295 6.831110e-03

ArmsTreatment_2 -384.5594 70.80549 42 -5.431209 2.599764e-06

ArmsTreatment_3 -544.2332 71.24447 42 -7.638953 1.797455e-09

ArmsTreatment_4 -316.3781 70.80549 42 -4.468271 5.860778e-05

ArmsTreatment_5 -664.9219 70.80549 42 -9.390824 7.079462e-12

Besides the above parametric statistical analysis, the corresponding nonparametric statistical analysis methods of one-way ANOVA and two-way ANOVA, Kruskal-Wallis test and Scheirer-Ray-Hare test, are provided to analyze the tumor volume of end point and the tumor growth rate. The output of method is as following.

DRAnalysis(oneAN.volume.data, pattern = 'oneAN', method = 'GR.KW')

Kruskal-Wallis rank sum test

data: growth.rate by Arms

Kruskal-Wallis chi-squared = 40.908, df = 5, p-value = 9.792e-08

#### 3.3.2 Permutation test

The permutation strategy is adopted to test whether significant difference in the tumor volume growth curves exists between different arms. This method is similar to the function of *compareGrowthCurves* in statmod package [23, 24]. For each pair of arms, DRAP first calculates t-statistics or Wilcox-statistics for each time point, and then calculates the mean of statistics among all time points. Subsequently, the animals in the arm pair are randomly allocated to two arms and the mean statistics was recalculated for 1000 times. The P value is the proportion of permutations where the mean statistics is greater in absolute value than the mean statistics for the original data set. Each pair of arms generates a P value. At last, the P-values are adjusted based on multiple testing among all possible arm pairs. This method is implemented by *CompareGC* in DRAP.

compareGC(oneAN.volume.data,

compare.to = 'neg.control',

neg.control = 'Control',

n = 1000,

fun = MeanW)

Arm1 Arm2 Stat p.value q.value

1 Treatment_1 Control 49.750 0.004 0.008

2 Treatment_2 Control 53.500 0.000 0.000

3 Treatment_3 Control 57.000 0.000 0.000

4 Treatment_4 Control 52.750 0.004 0.008

5 Treatment_5 Control 57.875 0.000 0.000

The result shows that the tumor volume growth curves of all treatments are significantly different with the *Control* arm.

### 3.4 TGI

Tumor growth inhibition (TGI) is one of the most generally used metrics to quantify the drug response of treatment arms compared to the control arm. The basic way to calculate TGI is following:

$TGI=\left（ 1-\frac{F(V_{T})}{F(V_{C})} \right）*100\%$ (1)

*F(V_T_)* and *F(V_C_)* mean the calculating ways for the treatment arm and control arm respectively. DRAP provides three types of *F( )* function to calculate TGI: (1) F=V_t_ - V_0_; (2) F=V_t_/V_0_; (3) F=area under the curve of tumor volume (AUC) [26]. V_t_ and V_0_ represent the mean tumor volume at the time t and time 0 respectively. It should be noted that TGI just suits to the experiment that measuring time point are consistent. For example, if F=V_t_ - V_0_, the TGI is expression as:

$TGI=\left（ 1-\frac{V_{T,t} - V_{T,0}}{V_{C,t} - V_{C,0}} \right）*100\%$ (2)

Calculating TGI is implemented by function *TGI*. The three methods are all included in this function.

oneAN.tgi <- TGI(data = oneAN.volume.data,

neg.control = 'Control',

method = 'AUC',

pattern = 'oneAN')

oneAN.tgi[1:10,]

Arms Times TGI

1 Treatment_1 3 0.1434423

2 Treatment_1 6 0.1449918

3 Treatment_1 9 0.1685218

4 Treatment_1 12 0.2165391

5 Treatment_1 15 0.2486146

6 Treatment_1 18 0.2549355

7 Treatment_1 21 0.2564916

8 Treatment_2 3 0.4133369

9 Treatment_2 6 0.3491789

10 Treatment_2 9 0.3407801

For presenting the result of TGI, DRAP offers two options. One is to present TGI value at the end of experiment (Figure S8); the other is to represent TGI value at all timepoints (Figure S9).

plotTGI(oneAN.tgi, pattern = 'oneAN', scope = 'end.point')


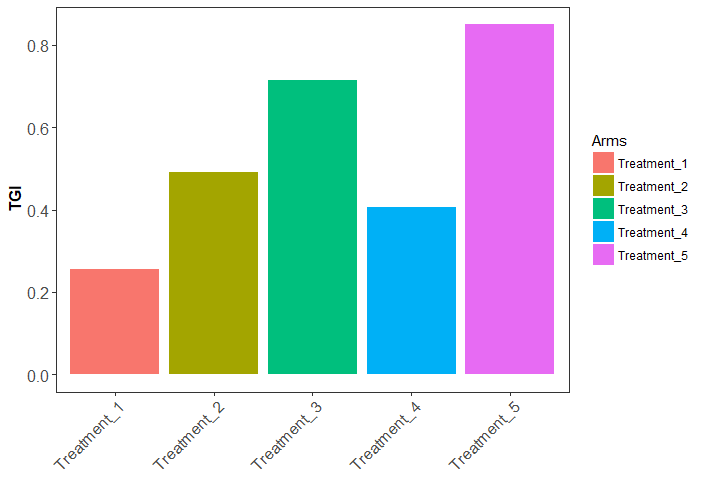


Figure S8. The presentation of TGI value at the end of experiment.

plotTGI(oneAN.tgi, pattern = 'oneAN', scope = 'all.point')


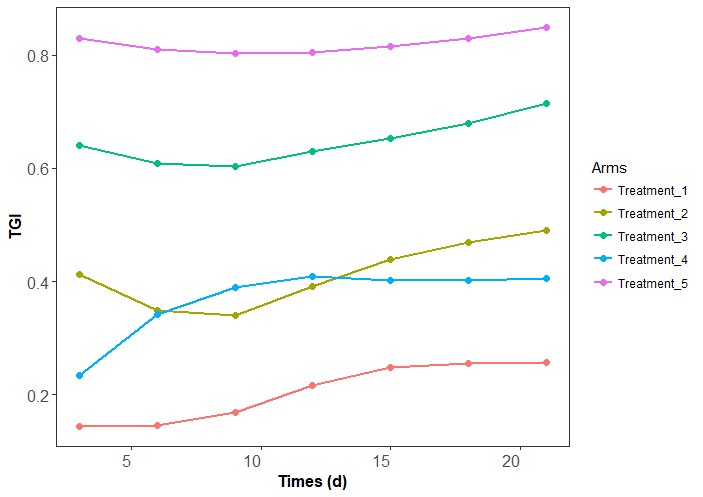


Figure S9. The presentation of TGI value at all time points.

The TGI value could rank the drug efficacy of all treatments for the patient. The result shows that the drug with best efficacy is *Treatment_5*.

### 3.5 Response level analysis

Drug response level is calculated to label the drug response of each animal, such as complete response (CR), partial response (PR), stable disease (SD) and progressive disease (PD) [8, 11, 12, 27]. The level is defined according to the tumor volume change after treatment. We implemented three standards to label drug response level: the one built in Novartis Institutes for BioMedical Research PDX encyclopedia (*NPDXE.Response*) [8], the one in Pediatric Preclinical Testing Program (*PPTP.Response*) [20, 28], and the one based on the relative change of tumor volumes (*RC.Response*) [12]. The details of the three methods are following.

*NPDXE.Response*: The response is determined by comparing tumor volume change at time t to its baseline: % tumor volume change = ∆Vol_t_ = 100% × ((V_t_ – V_initial_) / V_initial_). The BestResponse is the minimum value of ∆Vol_t_ for t ≥ 10 d. For each time t, the average of ∆Vol*_t_* from t = 0 to t is also calculated. The BestAvgResponse is defined as the minimum value of this average for t ≥ 10 d. This metric captures a combination of speed, strength and durability of response into a single value. The criteria for response is defined as follows (applied in this order): CR, BestResponse < -95% and BestAvgResponse < -40%; PR, BestResponse < -50% and BestAvgResponse < -20%; SD, BestResponse < 35% and BestAvgResponse < 30%; PD, not otherwise categorized.

The criteria of *NPDXE.Response* is organized in the following form:

npdxe.criteria <- data.frame(

BestResponse.lower = c(-1000,-0.95,-0.5,0.35),

BestResponse.upper = c(-0.95,-0.5,0.35,1000),

BestAvgResponse.lower = c(-1000,-0.4,-0.2,0.3),

BestAvgResponse.upper = c(-0.4,-0.2,0.3,1000),

Level = c('CR','PR', 'SD','PD'))

*PPTP.Response*: PD is defined as <50% regression from initial volume during the study period and >25% increase in initial volume at the end of study period. SD is defined as <50% regression from initial volume during the study period and 25% increase in initial volume at the end of the study. PR is defined as a tumor volume regression 50% for at least one time point but with measurable tumor (0.10 cm^3^). CR is defined as a disappearance of measurable tumor mass (<0.10 cm^3^) for at least one time point. In DRAP, we do not distinguish maintained CR (MCR) from CR and divide PD into PD1 or PD2 as in primary method.

The criteria of *PPTP.Response* is organized in the following form:

pptp.criteria <- data.frame(

min.RC.lower = c(-1,-1,-0.5,-0.5),

min.RC.upper = c(-0.5,-0.5,1000,1000),

min.Vol.lower = c(0,100,NA,NA),

min.Vol.upper = c(100,10000,NA,NA),

end.RC.lower =c(NA,NA,-1,0.25),

end.RC.upper = c(NA,NA,0.25,1000),

Level = c('CR','PR', 'SD','PD'))

*RC.Response*: Response levels are classified as follows: (1) CR, tumour regression with a decrease of at least 35% in tumour volume ; (2) PD, disease progression with at least a 35% increase in tumour volume; (3) SD, disease stabilization with a tumour graft volume at levels , 35% growth and , 35% regression.

The criteria of *RC.Response* is organized in the following form:

rc.criteria <- data.frame(RC.lower = c(-1000,-0.35,0.35),

RC.upper = c(-0.35,0.35,1000),

Level = c('CR','SD','PD'))

The three methods are implemented by *DRLevel*. Considering that tumor growth is influenced by the strains of mice, DRAP enables users to adjust the standards for defining response levels according to the experimental data and practical needs. Then we use *NPDXE.Response* to define response level of each animal as example to illustrate response level analysis.

oneAN.drl <- DRLevel(data = oneAN.volume.data,

method = 'NPDXE.Response',

criteria = npdxe.criteria,

neg.control = 'Control')

Warning message:

In DRLevel(data = oneAN.volume.data, method = "NPDXE.Response", :

The 'Reference' for suitable condition of NPDXE response criteria is: The initial tumor volume is about 200 mm3.

oneAN.drl <- oneAN.drl[order(oneAN.drl$Arms), ]

head(oneAN.drl)

ID Arms Best.Response Best.Avg.Response Response.Level

6 mouse_06 Treatment_1 2.720238 1.374603 PD

7 mouse_07 Treatment_1 3.044401 1.475974 PD

11 mouse_11 Treatment_1 2.546496 1.327986 PD

28 mouse_28 Treatment_1 2.447087 1.355054 PD

32 mouse_32 Treatment_1 2.860362 1.588893 PD

37 mouse_37 Treatment_1 2.246797 1.266390 PD

After labeling response level of each animal, *DRLevelSummary* exports summary of response level of each arm. The result is presented by *plotDRLevel* (Figure S10).

DRLevelSummary(oneAN.drl, by='Arms')

Arms Number Level Frequency Proportion

1 Treatment_1 8 PD 8 1.000

2 Treatment_2 8 PD 8 1.000

3 Treatment_3 8 PD 6 0.750

4 Treatment_3 8 SD 2 0.250

5 Treatment_4 8 PD 8 1.000

6 Treatment_5 8 PD 5 0.625

7 Treatment_5 8 SD 3 0.375

plotDRLevel(data = oneAN.drl, by='Arms', pattern = 'oneAN')


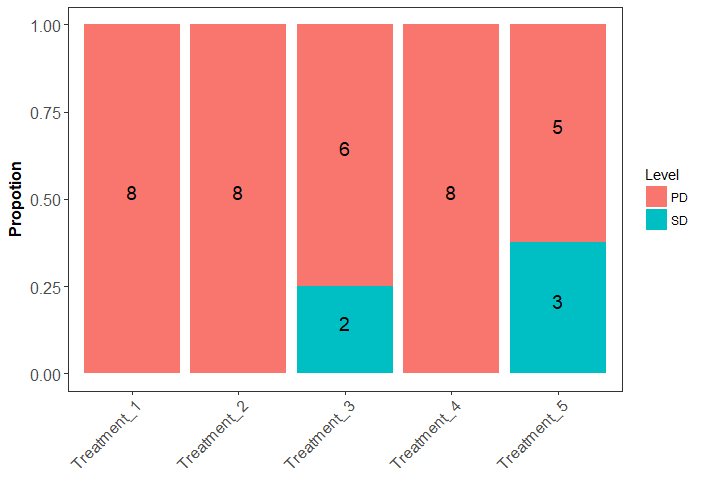


Figure S10. The representation of drug response level of each arm.

At last, the response evaluation indexes for each arm, were calculated, such as response rate (RR) and disease control rate (DCR). RR is the proportion of CR and PR among all testing objects in one arm, and DCR is the proportion of CR, PR and SD. response evaluation indexes, such as response rate (RR) and disease control rate (DCR). The results can be used to rank the drug efficacy of different arms. This function is implement by *DRLevelAnalysis*.

DRLevelAnalysis(oneAN.drl, by = 'Arms', measurement = 'DCR')

Arms Number DCR

1 Treatment_1 8 0.000

2 Treatment_2 8 0.000

3 Treatment_3 8 0.250

4 Treatment_4 8 0.000

5 Treatment_5 8 0.375

The result shown that 3 animals have obvious response to *Treatment_5*. This indicates that *Treatment_5* may have wide response for different subclones and show better response for the patient.

## 4. T*1*N pattern

This pattern aims to evaluate the anti-tumor efficacy of a particular treatment by using a PDX collection [12, 18]. Since a collection of xenografts are included, the effect of inter-tumor heterogeneity on drug response is sufficiently taken into account. Following the common protocol in preclinical data analysis, the drug response of each tumor line is calculated based on the mean or median of tumor volume values. The setting of multiple animals enrolled in one experimental group helps to increase the accuracy of response level of the tumor, and therefore acquire more precise evaluation of drug efficacy [15].

For this pattern, DRAP first defines response level of each tumor with the methods illustrated in *1*A*N* pattern, then calculates RR or DCR. Finally, DRAP uses waterfall plot to present the response level of every tumor (Figure 13), which provides an ease of visualization and interpretation [29]. The most representative example of *T*1*N* pattern is the work that tests response to EGFR blockade in a collection of colorectal cancers [12].

## 5. T*A*1 pattern

This pattern is designed for the high-throughput evaluation of a panel of treatment arms [8]. Similar to the above *T*1*N* pattern, a collection of xenografts are included, therefore inter-tumor heterogeneity could be taken into account. As *T*A*1* pattern involves a collection of tumors and a panel of treatment arms in one trial, this setting enrolls only one animal in each arm of every tumor line in order to balance costs with outcomes. The performance of this setting has been approved by an independent report [20].

The trial of this pattern could rank the drug efficacy of arms in in a special kind of tumor, evaluate the drug efficacy of a special arm in different kinds of tumors or in a special kind of tumor.

### 5.1 Input data

We use the dataset derived from Novartis Institutes for BioMedical Research PDX encyclopedia (NIBR PDXE) as example to introduce the function of DRAP for *T*A*1* pattern[8]. The dataset includes both tumor volume and body weight data for 6 tumor types, 277 tumors, and total 4771 animals responded to 61 treatments. The dataset in DRAP includes information “*Tumor*” “*ID*” “*Type*” “*Arms*” “*Times*” “*Volume*”.

data(TAone.volume.data)

head(TAone.volume.data)

Tumor ID Type Arms Times Volume

1 X-007 1 GC BGJ398 0 202.3

2 X-007 1 GC BGJ398 4 590.3

3 X-007 1 GC BGJ398 7 796.3

4 X-007 1 GC BGJ398 11 1004.5

5 X-007 2 GC BKM120 0 288.8

6 X-007 2 GC BKM120 4 419.9

### 5.2 Response level analysis

The response level of every animal is labeled by the method NPDXE.Response. Based on the response level of each animal, multiple purposes can be realized, including ranking drug efficacy of all arms in a special type of tumor, evaluating drug efficacy of a special arm in different types of tumors or in a special type of tumor. DRAP offers analysis and visualization tools for these purposes.

pdxe.criteria <- data.frame(

BestResponse.lower = c(-1000,-0.95,-0.5,0.35),

BestResponse.upper = c(-0.95,-0.5,0.35,1000),

BestAvgResponse.lower = c(-1000,-0.4,-0.2,0.3),

BestAvgResponse.upper = c(-0.4,-0.2,0.3,1000),

Level = c('CR','PR', 'SD','PD'))

TAone.drl <- DRLevel(data = TAone.volume.data,

criteria = npdxe.criteria,

method = 'NPDXE.Response',

neg.control = 'untreated')

Warning message:

In DRLevel(data = oneAN.volume.data, method = "NPDXE.Response", :

The 'Reference' for suitable condition of NPDXE response criteria is: The initial tumor volume is about 200 mm3.

head(TAone.drl)

ID Patient Type Arms Best.Response Best.Avg.Response Response.Level

1 1 X-007 GC BGJ398 3.96539792 2.20489372 PD

2 2 X-007 GC BKM120 1.43247922 0.77051593 PD

3 3 X-007 GC BYL719 3.03713142 1.96186749 PD

4 4 X-007 GC BYL719 + HSP990 0.02932151 0.08722582 SD

5 5 X-007 GC BYL719 + LJM716 0.39981826 0.40617901 PD

6 6 X-007 GC CLR457 0.25033171 0.26544302 SD

TAone.drl.summary <- DRLevelSummary(data = TAone.drl, by = 'Arms')

head(TAone.drl.summary)

Arms Number Level Frequency Proportion

1 5FU 43 PD 16 0.37209302

2 5FU 43 PR 1 0.02325581

3 5FU 43 SD 26 0.60465116

4 abraxane 37 PD 31 0.83783784

5 abraxane 37 PR 1 0.02702703

6 abraxane 37 SD 5 0.13513514

#### 5.2.1 Ranking drug efficacy of all arms in one type of tumor

The data of colorectal cancer (CRC) in NIBR PDXE was used to illustrates the functions of DRAP of ranking drug efficacy of all arms in a special type of tumor. DRAP ranks and presents the drug efficacy of all arms for CRC as in Figure 3A. The results showed that the combination of BYL719 and Binimetinib has the best efficacy, with RR being 28.57% and DCR being 85.71%. BYL719 is a selective inhibitor of PI3Kalpha [30]. LJM716 is an antibody drug targeted HER3 [31]. The information could help to determine the best candidate drugs or drug combinations for CRC in clinical trial.

crc.drl <- TAone.drl[TAone.drl$Type == 'CRC',]

crc.drl.summary <- DRLevelSummary(data = crc.drl, by='Arms')

head(crc.drl.summary)

Arms Number Level Frequency Proportion

1 5FU 43 PD 16 0.37209302

2 5FU 43 PR 1 0.02325581

3 5FU 43 SD 26 0.60465116

4 abraxane 1 PD 1 1.00000000

5 binimetinib 42 PD 14 0.33333333

6 binimetinib 42 PR 1 0.02380952

crc.drl.analysis <- DRLevelAnalysis(data = crc.drl, by = 'Arms', measurement = 'both')

crc.drl.analysis <- subset(crc.drl.analysis, Number >= 10)

crc.drl.analysis <- crc.drl.analysis[order(crc.drl.analysis$RR, decreasing = T),]

head(crc.drl.analysis)

Arms Number RR DCR

10 BYL719 + binimetinib 42 0.28571429 0.8571429

12 BYL719 + cetuximab + encorafenib 42 0.19047619 0.5714286

14 BYL719 + LJM716 42 0.11904762 0.7142857

15 cetuximab 43 0.11627907 0.4186047

11 BYL719 + cetuximab 41 0.09756098 0.6097561

16 cetuximab + encorafenib 42 0.09523810 0.5000000

plotDRResults(crc.drl, by = 'Arms', measurement = 'both', min.number = 10)


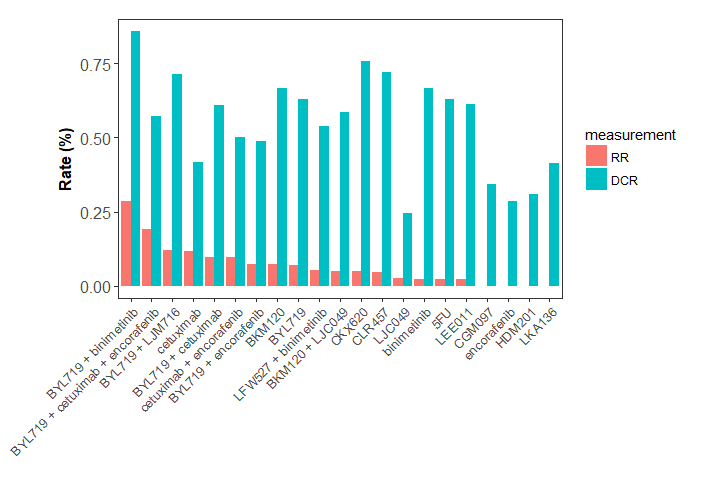


Figure S11. The drug efficacy of all arms in CRC.

#### 5.2.2 Evaluating drug efficacy of one arm in all types of tumors

For a new treatment, the question most frequently encountered is how to choose the preferred indication for clinical trial. To solve this question with PDXs, drug efficacy of the new treatment in different types of tumors should be evaluated and ranked. This part illustrates this function of DRAP with the data of different types of tumors respond to the combination of BYL719 and LJM716 in NIBR PDXE. Among the six tested tumor types, this treatment shown highest efficacy in gastric cancer (GC) (Figure S12). Based on this information, the developers could choose GC as the preferred indication for this new treatment in clinical trial.

bl.drl <- TAone.drl[TAone.drl$Arms == "BYL719 + LJM716",]

bl.drl.summary <- DRLevelSummary(data = bl.drl, by='Type')

head(bl.drl.summary)

Type Number Level Frequency Proportion

1 BRCA 39 CR 2 0.05128205

2 BRCA 39 PD 5 0.12820513

3 BRCA 39 PR 3 0.07692308

4 BRCA 39 SD 29 0.74358974

5 CRC 42 PD 12 0.28571429

6 CRC 42 PR 5 0.11904762

bl.drl.analysis <- DRLevelAnalysis(data=bl.drl, by='Type', measurement = 'both')

bl.drl.analysis[order(bl.drl.analysis$RR, decreasing = T),]

Type Number RR DCR

1 GC 58 0.22413793 0.6379310

5 NSCLC 27 0.22222222 0.5925926

4 BRCA 39 0.12820513 0.8717949

2 CRC 42 0.11904762 0.7142857

3 PDAC 34 0.05882353 0.7352941

plotDRResults(bl.drl, by = 'Type', measurement = 'both', min.number = 10)


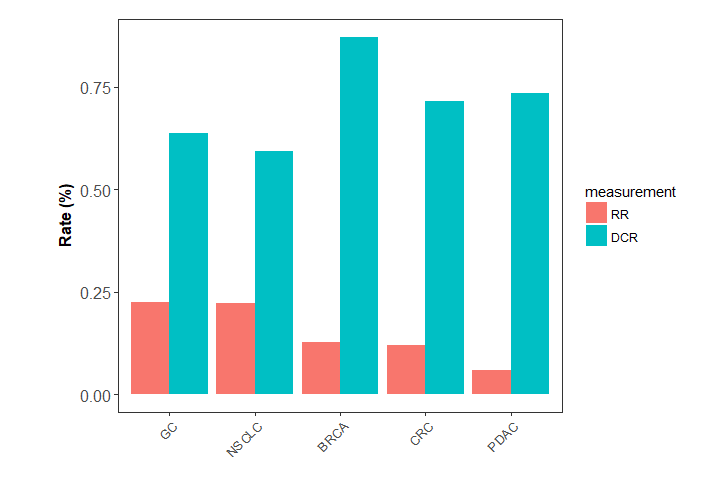


Figure S12. The drug efficacy of the combination of BYL719 and LJM716 in six different cancer types.

#### 5.2.3 Evaluating drug efficacy of one arm in one type of tumor

While evaluating drug efficacy of a special treatment in a particular type of tumor, waterfall plots is efficient way to represent the response level of individual animal, which provide an ease of visualization and interpretation [29]. This part illustrates this function of DRAP with the data of GC respond to the combination of BYL719 and LJM716 in NIBR PDXE. As in Figure S13, we could easily get the information of drug efficacy for each animal, how many animals in each response level, and the total drug efficacy in all samples.

bl.gc.drl <- bl.drl[bl.drl$Type == 'GC',]

plotWaterfall(data = bl.gc.drl, max.threshold = 100, order.by='Best.Response')


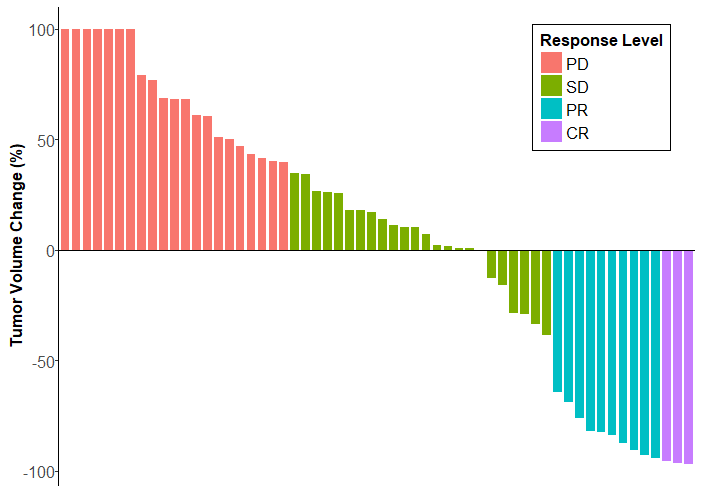


Figure S13. The waterfall plot of each animal response to the combination of BYL719 and LJM716 in GC.

## 6. T*A*N pattern

This pattern could be regarded as extended versions of the above three patterns, and could be applied in various situations. While applied to evaluate drug efficacy of multiple treatment arms, the analysis is consistent with *T*1*N* pattern [21]. While applied to evaluate drug response of multiple tumors, the analysis is similar to *1*A*N* pattern [11]. Of note, since this pattern includes multiple tumors in one trial and multiple animals in each arm, it allows for the investigation of both inter-tumor heterogeneity and intra-tumor heterogeneity.

At here, we used an example to illustrate the function of DRAP for evaluating drug response of multiple tumors. The result reflects the heterogeneity of drug response in different tumors. The example for evaluating drug efficacy of multiple treatment arms can be found in the publication [21].

### 6.1 Input data

Besides the requirements of input data for *1*A*N pattern*, the input data of DRAP for *T*A*N pattern* needs additional information of “*Tumor*”. The example data for this pattern includes four tumors and four treatments, and is processed as in *1*A*N pattern*. The example is following.

data(TAN.volume.data)

TAN.volume.data[1:10,]

Arms Tumor ID Times Volume

1 Control Tumor_1 mouse_47 0 238.0

2 Control Tumor_1 mouse_47 3 561.5

3 Control Tumor_1 mouse_47 6 653.7

4 Control Tumor_1 mouse_47 9 1088.8

5 Control Tumor_1 mouse_47 12 1475.2

6 Control Tumor_1 mouse_47 15 1517.7

7 Control Tumor_1 mouse_47 18 1852.2

8 Control Tumor_1 mouse_47 21 2046.6

9 Control Tumor_1 mouse_55 0 223.3

10 Control Tumor_1 mouse_55 3 424.8

### 6.2 Data presentation and analysis

The presentation of tumor volume data and body weight data, calculation and visualization of TGI, and statistical analysis for *T*A*N* pattern in DARP is similar to *1*A*N* pattern.

While presenting the tumor volume data or body weight data at the level of *Animal*, the figure is organized in form of matrix graph with each element representing the data of one tumor line in one arm (Figure S14). While presenting the tumor volume data or body weight data at the level of *Arm*, the figure is grouped by *Tumor* (Figure S15).

plotVolumeGC(TAN.volume.data, level = 'Animal', pattern = 'TAN')


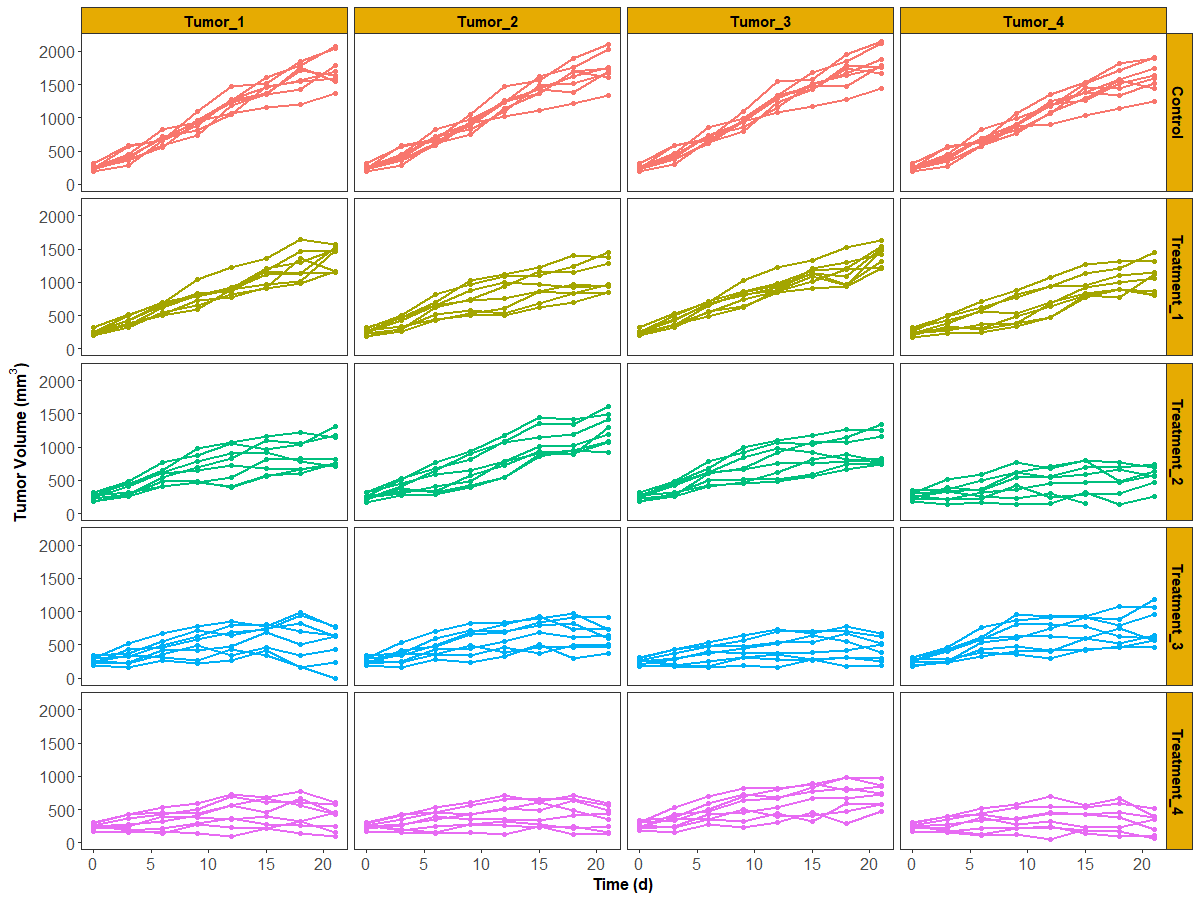


Figure S14. The presentation of tumor volume data at the level of *Animal* for *T*A*N* pattern.

plotVolumeGC(TAN.volume.data, level = 'Arm', pattern = 'TAN', position.dodge = 0.5)


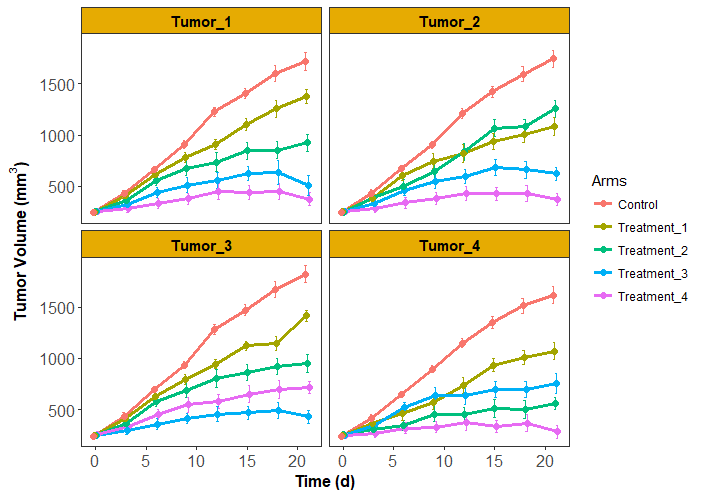


Figure S15. The presentation of tumor volume data at the level of *Arm* for *T*A*N* pattern.

In the analysis part, *P*A*N pattern* includes another factor *Tumor*, so the ANOVA analysis is replaced by two-ways ANOVA.

DRAnalysis(TAN.volume.data, pattern = 'TAN', method = 'endpoint.ANOVA')

Df Sum Sq Mean Sq F value Pr(>F)

Arms 4 33958717 8489679 182.003 < 2e-16 ***

Patient 3 849129 283043 6.068 0.000655 ***

Arms:Patient 12 3297251 274771 5.891 3.36e-08 ***

Residuals 138 6437123 46646

---

Signif. codes: 0 ‘***’ 0.001 ‘**’ 0.01 ‘*’ 0.05 ‘.’ 0.1 ‘ ’ 1

The nonparametric analysis is replaced by Scheirer-Ray-Hare test, which is the corresponding nonparametric method of two-ways ANOVA.

DRAnalysis(TAN.volume.data, pattern = 'TAN', method = 'endpoint.SRH')

DV: Volume

Observations: 158

D: 0.9999985

MS total: 2093.5

Df Sum Sq H p.value

Arms 4 250765 119.783 0.00000

Patient 3 6489 3.100 0.37649

Arms:Patient 12 27958 13.354 0.34381

Residuals 138 43467

The calculating of TGI is same as in *1*A*N* pattern. The presentation of TGI is grouped by *Tumor* (Figure S16).

TAN.tgi <- TGI(TAN.volume.data, neg.control = 'Control', pattern = 'TAN',method = 'AUC')

plotTGI(TAN.tgi,pattern = 'TAN',scope = 'all.point')


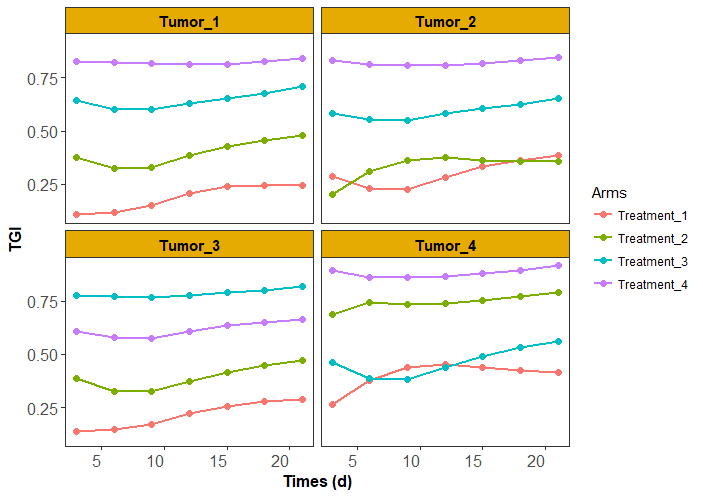


Figure S16. The presentation of TGI at the scope of *all.point* for *T*A*N* pattern.

### 6.3 Response level analysis

The drug response level analysis for this pattern is similar to *1*A*N* pattern. The presentation of response level is implemented at the level of *Arm* (Figure S17) or at the level of *Arm* and *Tumor* (Figure S18).

TAN.drl <- DRLevel(TAN.volume.data,

criteria = npdxe.criteria,

method = 'NPDXE.Response',

neg.control = 'Control')

Warning message:

In DRLevel(data = oneAN.volume.data, method = "NPDXE.Response", :

The 'Reference' for suitable condition of NPDXE response criteria is: The initial tumor volume is about 200 mm3.

head(TAN.drl)

ID Arms Patient Best.Response Best.Avg.Response Response.Level

1 mouse_1 Treatment_4 Patient_2 1.1105263 0.7187970 PD

2 mouse_10 Treatment_4 Patient_1 1.1657895 0.6894737 PD

3 mouse_100 Treatment_4 Patient_3 0.5724401 0.2592593 PD

4 mouse_101 Treatment_3 Patient_4 1.1634512 0.6703108 PD

6 mouse_103 Treatment_3 Patient_3 0.7495301 0.5477444 PD

9 mouse_106 Treatment_1 Patient_2 2.2502680 1.1674169 PD

DRLevelSummary(TAN.drl, by='Arms')

Arms Number Level Frequency Proportion

1 Treatment_1 32 PD 32 1.00000

2 Treatment_2 32 PD 29 0.90625

3 Treatment_2 32 SD 3 0.09375

4 Treatment_3 32 PD 25 0.78125

5 Treatment_3 32 SD 7 0.21875

6 Treatment_4 32 PD 21 0.65625

7 Treatment_4 32 PR 3 0.09375

8 Treatment_4 32 SD 8 0.25000

DRLevelAnalysis(TAN.drl, by='Arms', measurement = 'both')

Arms Number RR DCR

1 Treatment_1 32 0.00000 0.00000

2 Treatment_2 32 0.00000 0.09375

3 Treatment_3 32 0.00000 0.21875

4 Treatment_4 32 0.09375 0.34375

plotDRLevel(data = TAN.drl, by='Arms', pattern = 'TAN')


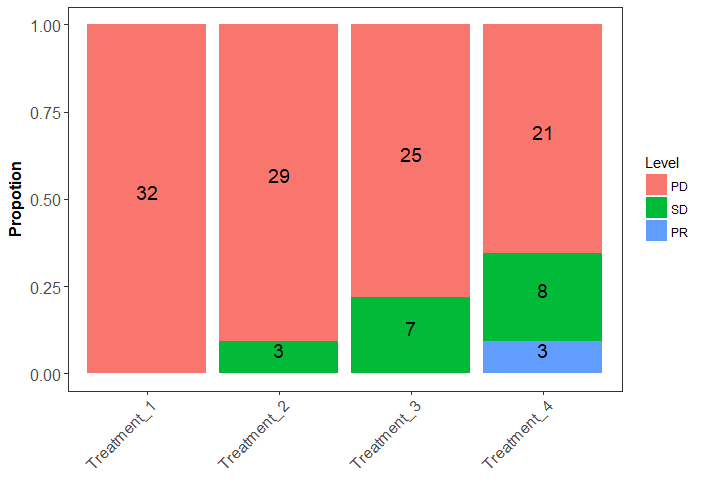


Figure S17. The presentation of response level for *P*A*N* pattern at the level of *Arm*.

Based on the result generated at the *Arm* level, it shows that *Treatment_4* possesses the widest treatment efficacy for the all patients.

DRLevelAnalysis(TAN.drl, by=c('Arms','Tumor'), measurement = 'both')

Arms Tumor Number RR DCR

1 Treatment_1 Tumor_1 8 0.000 0.000

2 Treatment_1 Tumor_2 8 0.000 0.000

3 Treatment_1 Tumor_3 8 0.000 0.000

4 Treatment_1 Tumor_4 8 0.000 0.000

5 Treatment_2 Tumor_1 8 0.000 0.000

6 Treatment_2 Tumor_2 8 0.000 0.000

7 Treatment_2 Tumor_3 8 0.000 0.000

8 Treatment_2 Tumor_4 8 0.000 0.375

9 Treatment_3 Tumor_1 8 0.000 0.375

10 Treatment_3 Tumor_2 8 0.000 0.000

11 Treatment_3 Tumor_3 8 0.000 0.375

12 Treatment_3 Tumor_4 8 0.000 0.125

13 Treatment_4 Tumor_1 8 0.000 0.375

14 Treatment_4 Tumor_2 8 0.000 0.375

15 Treatment_4 Tumor_3 8 0.000 0.000

16 Treatment_4 Tumor_4 8 0.375 0.625

plotDRLevel(data = TAN.drl, by=c('Arms','Tumor'), pattern = 'TAN')


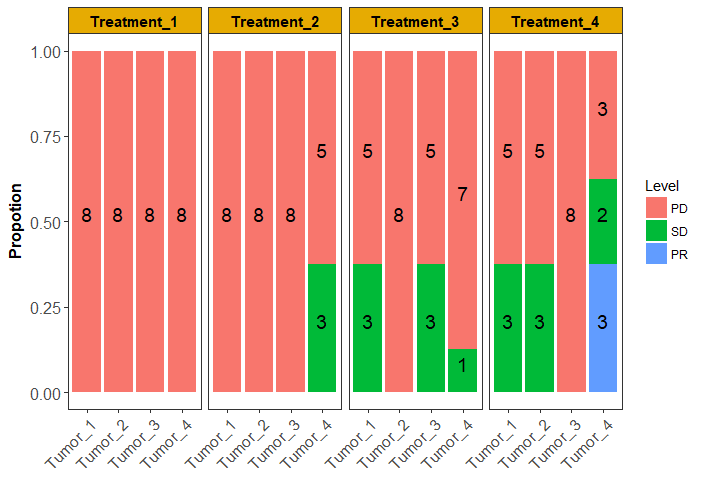


Figure S18. The presentation of response level at the level of *Arm* and *Tumor*.

Based on the results generated from both *Arm* and *Patient* level, it shows that the best treatment for *Patient_1*, *Patient_2* and *Patient_4* is *Treatment_4*, but for *Patient_3* is *Treatment_3*.

## Reference

1. Tentler JJ, Tan AC, Weekes CD, Jimeno A, Leong S, Pitts TM, Arcaroli JJ, Messersmith WA, Eckhardt SG: **Patient-derived tumour xenografts as models for oncology drug development.** *Nat Rev Clin Oncol* 2012, **9:**338-350.

2. Hidalgo M, Amant F, Biankin AV, Budinska E, Byrne AT, Caldas C, Clarke RB, de Jong S, Jonkers J, Maelandsmo GM, et al: **Patient-derived xenograft models: an emerging platform for translational cancer research.** *Cancer Discov* 2014, **4:**998-1013.

3. Kemper K, Krijgsman O, Cornelissen-Steijger P, Shahrabi A, Weeber F, Song JY, Kuilman T, Vis DJ, Wessels LF, Voest EE, et al: **Intra- and inter-tumor heterogeneity in a vemurafenib-resistant melanoma patient and derived xenografts.** *EMBO Mol Med* 2015, **7:**1104-1118.

4. DeRose YS, Wang G, Lin YC, Bernard PS, Buys SS, Ebbert MT, Factor R, Matsen C, Milash BA, Nelson E, et al: **Tumor grafts derived from women with breast cancer authentically reflect tumor pathology, growth, metastasis and disease outcomes.** *Nat Med* 2011, **17:**1514-1520.

5. Bruna A, Rueda OM, Greenwood W, Batra AS, Callari M, Batra RN, Pogrebniak K, Sandoval J, Cassidy JW, Tufegdzic-Vidakovic A, et al: **A Biobank of Breast Cancer Explants with Preserved Intra-tumor Heterogeneity to Screen Anticancer Compounds.** *Cell* 2016, **167:**260-274.e222.

6. Morton CL, Houghton PJ: **Establishment of human tumor xenografts in immunodeficient mice.** *Nat Protoc* 2007, **2:**247-250.

7. Byrne AT, Alferez DG, Amant F, Annibali D, Arribas J, Biankin AV, Bruna A, Budinska E, Caldas C, Chang DK, et al: **Interrogating open issues in cancer precision medicine with patient-derived xenografts.** *Nat Rev Cancer* 2017, **17:**254-268.

8. Gao H, Korn JM, Ferretti S, Monahan JE, Wang Y, Singh M, Zhang C, Schnell C, Yang G, Zhang Y, et al: **High-throughput screening using patient-derived tumor xenografts to predict clinical trial drug response.** *Nat Med* 2015, **21:**1318-1325.

9. Owonikoko TK, Zhang G, Kim HS, Stinson RM, Bechara R, Zhang C, Chen Z, Saba NF, Pakkala S, Pillai R, et al: **Patient-derived xenografts faithfully replicated clinical outcome in a phase II co-clinical trial of arsenic trioxide in relapsed small cell lung cancer.** *J Transl Med* 2016, **14:**111.

10. Wu X, Zhang J, Zhen R, Lv J, Zheng L, Su X, Zhu G, Gavine PR, Xu S, Lu S, et al: **Trastuzumab anti-tumor efficacy in patient-derived esophageal squamous cell carcinoma xenograft (PDECX) mouse models.** *J Transl Med* 2012, **10:**180.

11. Stewart E, Federico SM, Chen X, Shelat AA, Bradley C, Gordon B, Karlstrom A, Twarog NR, Clay MR, Bahrami A, et al: **Orthotopic patient-derived xenografts of paediatric solid tumours.** *Nature* 2017, **549:**96-100.

12. Bertotti A, Papp E, Jones S, Adleff V, Anagnostou V, Lupo B, Sausen M, Phallen J, Hruban CA, Tokheim C, et al: **The genomic landscape of response to EGFR blockade in colorectal cancer.** *Nature* 2015, **526:**263-267.

13. Aparicio S, Hidalgo M, Kung AL: **Examining the utility of patient-derived xenograft mouse models.** *Nat Rev Cancer* 2015, **15:**311-316.

14. Day CP, Merlino G, Van Dyke T: **Preclinical mouse cancer models: a maze of opportunities and challenges.** *Cell* 2015, **163:**39-53.

15. Guo S, Mao B, Li H: **Theory and methodology for the design and analysis of PDX mouse clinical trials.** *Cancer Res* 2017, **77:**4534-4534.

16. Morelli MP, Calvo E, Ordonez E, Wick MJ, Viqueira BR, Lopez-Casas PP, Bruckheimer E, Calles-Blanco A, Sidransky D, Hidalgo M: **Prioritizing phase I treatment options through preclinical testing on personalized tumorgraft.** *J Clin Oncol* 2012, **30:**e45-48.

17. Bertotti A, Migliardi G, Galimi F, Sassi F, Torti D, Isella C, Cora D, Di Nicolantonio F, Buscarino M, Petti C, et al: **A molecularly annotated platform of patient-derived xenografts ("xenopatients") identifies HER2 as an effective therapeutic target in cetuximab-resistant colorectal cancer.** *Cancer Discov* 2011, **1:**508-523.

18. Zhang L, Yang J, Cai J, Song X, Deng J, Huang X, Chen D, Yang M, Wery JP, Li S, et al: **A subset of gastric cancers with EGFR amplification and overexpression respond to cetuximab therapy.** *Sci Rep* 2013, **3:**2992.

19. Migliardi G, Sassi F, Torti D, Galimi F, Zanella ER, Buscarino M, Ribero D, Muratore A, Massucco P, Pisacane A, et al: **Inhibition of MEK and PI3K/mTOR suppresses tumor growth but does not cause tumor regression in patient-derived xenografts of RAS-mutant colorectal carcinomas.** *Clin Cancer Res* 2012, **18:**2515-2525.

20. Murphy B, Yin H, Maris JM, Kolb EA, Gorlick R, Reynolds CP, Kang MH, Keir ST, Kurmasheva RT, Dvorchik I, et al: **Evaluation of Alternative In Vivo Drug Screening Methodology: A Single Mouse Analysis.** *Cancer Res* 2016, **76:**5798-5809.

21. Li J, Ye C, Mansmann UR: **Comparing Patient-Derived Xenograft and Computational Response Prediction for Targeted Therapy in Patients of Early-Stage Large Cell Lung Cancer.** *Clin Cancer Res* 2016, **22:**2167-2176.

22. Krepler C, Xiao M, Sproesser K, Brafford PA, Shannan B, Beqiri M, Liu Q, Xu W, Garman B, Nathanson KL, et al: **Personalized Preclinical Trials in BRAF Inhibitor-Resistant Patient-Derived Xenograft Models Identify Second-Line Combination Therapies.** *Clin Cancer Res* 2016, **22:**1592-1602.

23. Castro MA, de Santiago I, Campbell TM, Vaughn C, Hickey TE, Ross E, Tilley WD, Markowetz F, Ponder BA, Meyer KB: **Regulators of genetic risk of breast cancer identified by integrative network analysis.** *Nat Genet* 2016, **48:**12-21.

24. Elso CM, Roberts LJ, Smyth GK, Thomson RJ, Baldwin TM, Foote SJ, Handman E: **Leishmaniasis host response loci (lmr1-3) modify disease severity through a Th1/Th2-independent pathway.** *Genes Immun* 2004, **5:**93-100.

25. Cassidy JW, Caldas C, Bruna A: **Maintaining Tumor Heterogeneity in Patient-Derived Tumor Xenografts.** *Cancer Res* 2015, **75:**2963-2968.

26. Hoeflich KP, Merchant M, Orr C, Chan J, Den Otter D, Berry L, Kasman I, Koeppen H, Rice K, Yang NY, et al: **Intermittent administration of MEK inhibitor GDC-0973 plus PI3K inhibitor GDC-0941 triggers robust apoptosis and tumor growth inhibition.** *Cancer Res* 2012, **72:**210-219.

27. Stewart E, Goshorn R, Bradley C, Griffiths LM, Benavente C, Twarog NR, Miller GM, Caufield W, Freeman BB, 3rd, Bahrami A, et al: **Targeting the DNA repair pathway in Ewing sarcoma.** *Cell Rep* 2014, **9:**829-841.

28. Houghton PJ, Morton CL, Tucker C, Payne D, Favours E, Cole C, Gorlick R, Kolb EA, Zhang W, Lock R, et al: **The pediatric preclinical testing program: description of models and early testing results.** *Pediatr Blood Cancer* 2007, **49:**928-940.

29. Gengenbacher N, Singhal M, Augustin HG: **Preclinical mouse solid tumour models: status quo, challenges and perspectives.** *Nat Rev Cancer* 2017, **17:**751-765.

30. Fritsch C, Huang A, Chatenay-Rivauday C, Schnell C, Reddy A, Liu M, Kauffmann A, Guthy D, Erdmann D, De Pover A, et al: **Characterization of the novel and specific PI3Kalpha inhibitor NVP-BYL719 and development of the patient stratification strategy for clinical trials.** *Mol Cancer Ther* 2014, **13:**1117-1129.

31. Garner A, Sheng Q, Bialucha U, Chen D, Chen Y, Das R, Elis W, Heidt A, Kunz C, Li S, et al: **Abstract 2733: LJM716: an anti-HER3 antibody that inhibits both HER2 and NRG driven tumor growth by trapping HER3 in the inactive conformation.** *Cancer Research* 2012, **72:**2733.
